# Single Cell Stochastic Regulation of Pilus Phase Variation by an Attenuation-like Mechanism

**DOI:** 10.1371/journal.ppat.1003860

**Published:** 2014-01-16

**Authors:** Camille Danne, Sarah Dubrac, Patrick Trieu-Cuot, Shaynoor Dramsi

**Affiliations:** 1 Institut Pasteur, Unité de Biologie des Bactéries Pathogènes à Gram positif, Paris, France; 2 CNRS ERL 3526, Paris, France; 3 Université Paris Diderot-Sorbonne Paris Cité, Paris, France; Boston Children's Hospital, United States of America

## Abstract

The molecular triggers leading to virulence of a number of human-adapted commensal bacteria such as *Streptococcus gallolyticus* are largely unknown. This opportunistic pathogen is responsible for endocarditis in the elderly and associated with colorectal cancer. Colonization of damaged host tissues with exposed collagen, such as cardiac valves and pre-cancerous polyps, is mediated by appendages referred to as Pil1 pili. Populations of *S. gallolyticus* are heterogeneous with the majority of cells weakly piliated while a smaller fraction is hyper piliated. We provide genetic evidences that heterogeneous *pil1* expression depends on a phase variation mechanism involving addition/deletion of GCAGA repeats that modifies the length of an upstream leader peptide. Synthesis of longer leader peptides potentiates the transcription of the *pil1* genes through ribosome-induced destabilization of a premature stem-loop transcription terminator. This study describes, at the molecular level, a new regulatory mechanism combining phase variation in a leader peptide-encoding gene and transcription attenuation. This simple and robust mechanism controls a stochastic heterogeneous pilus expression, which is important for evading the host immune system while ensuring optimal tissue colonization.

## Author Summary

*Streptococcus gallolyticus* (formely known as *S. bovis* biotype I) is an emerging cause of septicemia and endocarditis in the elderly. Intriguingly, epidemiological studies revealed a strong association, up to 65%, between endocarditis due to *S. gallolyticus* and colorectal malignancies. Whether *S. gallolyticus* infection is a cause or a consequence of colon cancer remains to be investigated. We previously showed that colonization of damaged cardiac valves with exposed collagen is mediated by the Pil1 pilus in *S. gallolyticus*. In the present work, we report that Pil1 is heterogeneously expressed at the single cell level, giving rise to two distinct bacterial subpopulations, a majority of weakly piliated cells and a minority of hyper-piliated cells. We have characterized, at the molecular level, a novel regulatory mechanism responsible for Pil1 heterogeneous expression combining phase variation in the leader peptide and transcriptional attenuation. Pili are highly immunogenic proteins proposed as vaccine candidate in pathogenic streptococci whose expression involves a fitness cost due to the selective pressure of host immune responses. Hence, this robust and simple system mitigates susceptibility to immune defenses while ensuring optimal colonization of host tissues.

## Introduction

*Streptococcus gallolyticus*, formerly known as *Streptococcus bovis* biotype I, is present asymptomatically in the gastrointestinal tract of 2.5–15% of the human population [1]. However, this commensal bacterium can become a pathogen responsible for infective endocarditis in the elderly. Intriguingly, epidemiological studies pointed out a strong association, up to 65%, between endocarditis due to *S. gallolyticus* and colorectal malignancies [1]–[3]. Whether *S. gallolyticus* presence is a cause or a consequence of colon cancer development remains unknown [4]. Genome analysis of *S. gallolyticus* UCN34, a strain isolated from a patient suffering from infective endocarditis and colon cancer, revealed the existence of three pilus loci named *pil1*, *pil2*, and *pil3* [5]. Pili are long filamentous structures extending from the bacterial surface, composed of covalently linked pilin subunits, which play key roles in adhesion and colonization of host tissues. Each pilus locus encodes two structural LPXTG proteins and one sortase C, an enzyme which covalently links pilin subunits during assembly of the pilus filament. The Pil1 locus of strain UCN34 is composed of three genes encoding a major pilin, PilB (Gallo2178), a collagen-binding adhesin, PilA (Gallo2179), and a sortase C (Gallo2177). In previous studies, PilA was shown to bind to collagen type I, the major component of cardiac valves, and to collagen type IV, enriched in basal lamina of pre-cancerous polyps [6], [7]. PilA constitutes the major collagen-binding protein in *S. gallolyticus*, conferring adhesive properties to the pilus, and is involved in the development of infective endocarditis in a rat experimental model [7].

Using immunogold electron microscopy, we previously noted that expression of Pil1 pilus in *S. gallolyticus* strain UCN34 was heterogeneous with less than half of the bacteria expressing detectable pili [7]. Similar observations have been reported in other piliated gram-positive bacteria, such as *Corynebacterium renale* and *Corynebacterium pilosum*, *Streptococcus pneumoniae, Streptococcus pyogenes*, and *Enterococcus faecalis* [8]–[16]. Regulation of pilus genes expression occurs primarily at the transcriptional level and most pilus regulatory genes are located immediately upstream of the divergently transcribed pilus loci [17]. The corresponding regulators belong either to the AtxA/Mga superfamily (e.g. EbpR in *E. faecalis*), including the RALP-family of RofA-like regulators (RlrA in *S. pneumoniae*, Nra in *S. pyogenes* and RogB in *S. agalactiae*) or are members of the AraC/XylS- family (MsmR in *S. pyogenes* and Ape1 in *S. agalactiae*).

A bistability mechanism was proposed recently to explain the heterogeneous expression of the PI-1 pilus in *S. pneumoniae* [12], [18]. In this bacterium, the transcription of PI-1 locus is tightly controlled by RlrA, which also activates its own expression. The bistable expression of the PI-1 genes is mediated by this positive-feedback loop [19]. It was therefore proposed that cells in which *rlrA* expression is autoactivated display high intracellular concentrations of RlrA with the concomitant high-level expression of the pilus genes. Return to the low expressing pilus state is mediated by the pilus structural component RrgA acting as a negative regulator of RlrA [12]. External environmental factors could also influence the heterogeneous expression of pili. For example, in *S. pyogenes* M49, the percentage of cells expressing the FCT-3 pilus increased with lower temperatures, where it is significantly higher at the temperature of external surfaces such as skin (47% at 30°C) compared to the body temperature (20% at 37°C) [14]. In *E. faecalis* strain OG1RF, expression of the pilus operon *ebpABC* increased in presence of bicarbonate [15] or serum [20], with more cells producing pili, and the extent of piliation in bacteria recovered from rat endocarditis vegetations was even higher. Although the mechanisms explaining the various regulatory features of pilus expression are largely unknown, these observations highlight the fact that bacteria respond to physiological conditions by altering their tissue adhesive capacity.

In this study, we identified a new regulatory mechanism that controls heterogeneous expression of Pil1 in *S. gallolyticus* UCN34. We showed that the heterogeneity of pilus Pil1 expression depends on a phase variation mechanism between short tandem repeats in a leader peptide-encoding gene located at the 5′ end of the pilus gene cluster transcript. Some of these rearrangements provoke sequence frameshift leading to synthesis of longer leader peptides that potentiate transcription of the downstream *pil1* genes through ribosome-induced destabilization of a stem-loop transcription terminator. Pili are highly immunogenic protein polymers required for bacterial adhesion. Therefore, this stochastic mechanism of pilus expression constitutes a robust and simple system used by this organism to evade the host immune response and, when needed, to ensure optimal colonization of host tissues.

## Results

### Heterogeneous expression of Pil1 pilus in *S. gallolyticus* UCN34

**Figure 1:**
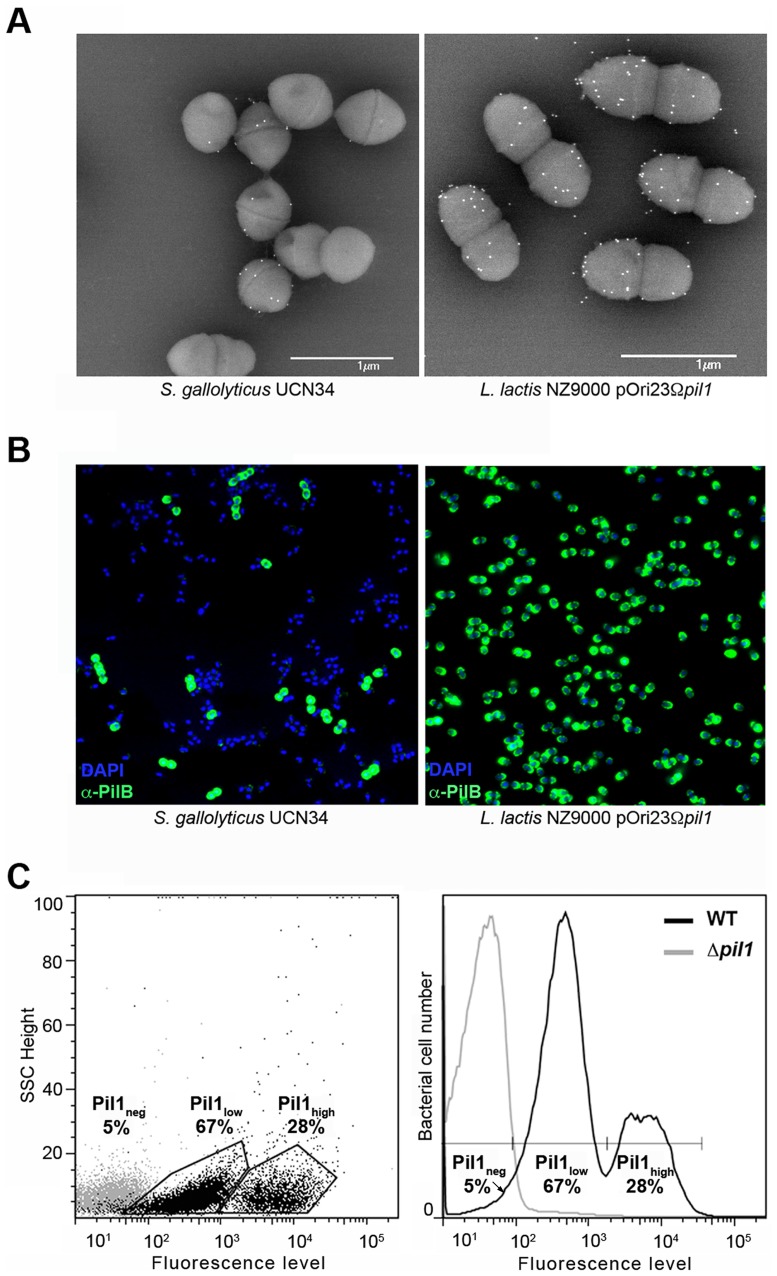
Heterogeneous expression of Pil1 pilus at the *Streptococcus gallolyticus* UCN34 surface. (A–B) Scanning immunogold electron microscopy and immunofluorescence of *S. gallolyticus* UCN34 strain and *L. lactis* NZ9000 pOri23Ω*pil1* strain expressing *pil1*. Pili were revealed with an anti-PilB polyclonal antibody (pAb) coupled to 10 nm gold beads (A) or the DyLight 488 rabbit secondary antibody (B). (C) Flow cytometry analysis of *S. gallolyticus* UCN34 cell populations labeled with anti-PilB pAb and the DyLight 488 rabbit secondary antibody. Results are represented as dot plots (left) or as a graph (right). The two groups (left) or peaks (right) in black correspond to the heterogeneous UCN34 WT strain constituted of a weakly piliated subpopulation (Pil1_low_ cells) and of a hyper piliated subpopulation (Pil1_high_ cells) representing 67% and 28% of the total population respectively. The negative population in gray corresponds to the isogenic Δ*pil1* mutant (Pil1_neg_).

Examination of immunogold scanning electron micrographs at lower magnification revealed a heterogeneous expression of Pil1 pilus in the *S. gallolyticus* strain UCN34, with less than 50% of the cells in the population displaying pili on their surface when labeled with a specific antibody against the major pilin PilB. This result contrasts with the homogeneous labeling of cells when the *pil1* operon is constitutively expressed in the heterologous host *Lactococcus lactis* NZ9000 (Fig. 1A). Consistently, two distinct subpopulations of cells with low (Pil1_low_) and high (Pil1_high_) Pil1 pilus levels were observed by immunofluorescence analyses with anti-PilB antibody whereas, again, no heterogeneity was seen in a lactococcal strain expressing *pil1* under the control of a constitutive promoter (Fig. 1B). Quantification of these data by flow cytometry analyses showed that Pil1_low_ cells account for 67% and Pil1_high_ cells for 28% of the total population; the remaining 5% being Pil1 negative (Fig. 1C and 1D). Similar results were obtained using antibodies against the pilus-associated adhesin PilA for immunodetection and flow cytometry (data not shown).

### Analysis of the *pil1* locus of *S. gallolyticus* UCN34

**Figure 2:**
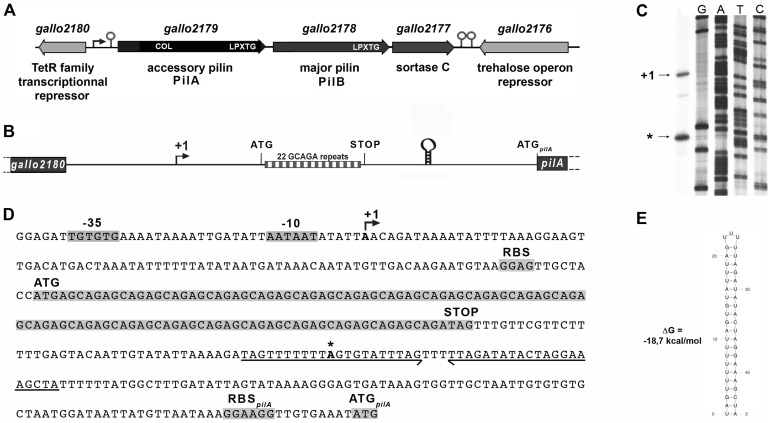
The *pil1* locus of *S. gallolyticus* strain UCN34. (A) Schematic representation of the *pil1* operon constituted of three genes: *pilA*, encoding the pilus adhesin, *pilB* encoding the major pilin and *srtC*, encoding the sortase required for pilus polymerization. Relevant domains are: COL, collagen-binding domain (Pfam 05737); and LPXTG, cell wall-anchoring domain. The P*pil1* promoter, the premature terminator upstream *pilA* and the putative terminator (hairpin structures) downstream *srtC* delineating the *pil1* operon are indicated. (B) Schematic representation of the intergenic region *gallo2180-pilA* with the 22 GCAGA repeats striped in gray and white and the stem-loop structure. (C) Determination of transcription start sites (TSS) of the *pil1* operon by primer extension analysis. The band indicated as “+1” corresponds to the location upstream the leader peptide gene is the likely TSS of the *pil1* operon; the band marked as “*” is located within one inverted repeat of the stem-loop structure and is likely generated by a pause of the RNA polymerase during elongation. (D) Nucleotide sequence of the intergenic region *gallo2180-pilA* showing the -35 and -10 promoter boxes and the +1 site, the leader peptide coding sequence with the 22 GCAGA repeats, the downstream stem-loop structure, the *pilA* initiator codon and the predicted ribosome binding site (RBS). (E) The predicted secondary structure of the stem-loop transcription terminator identified upstream ATG*_pilA_* (according to The mfold WebServer, http://mfold.rna.albany.edu/?q=mfold/RNA-Folding-Form).

The *pil1* locus of *S. gallolyticus* strain UCN34 consists of three genes encoding the collagen-binding adhesin PilA (Gallo2179), the major pilin PilB (Gallo2178), and the sortase C enzyme (Gallo2177) responsible for the covalent polymerization of PilA and PilB (Fig. 2A) [5], [7]. Upstream and divergent from *pil1* lies a gene, *gallo2180*, encoding a putative transcriptional regulator belonging to the TetR family (Fig. 2A). However, this regulatory gene is not always associated to the *pil1* locus in *S. gallolyticus* isolates. It is present in the genomes of all sequenced *S. gallolyticus* strains, even in those that do not carry the *pil1* locus, and in some closely related non-pathogenic species that lack this operon, such as *Streptococcus macedonicus*. Quantitative RT-PCR did not reveal any changes in the transcript levels of *gallo2180* in our set of clinical strains displaying different levels of *pil1* [7]. Finally, overexpression or mutational inactivation of *gallo2180* in strain UCN34 did not alter the pattern of Pil1 expression but strongly altered bacterial cell morphology (data not shown). Taken together, these results demonstrate that *gallo2180* gene product does not control *pil1* transcription.

A closer examination into the intergenic region located between *gallo2180* and pilA revealed the presence of 22 GCAGA repeats (110 nt) followed by a putative stem-loop structure (ΔG = −18.7 kcal/mol) (Fig. 2B, 2D and 2E). It is located entirely within a predicted open reading frame (ORF) specifying a putative short 38 amino acid peptide, with a recognizable ribosome binding side immediately upstream of an ATG. This remarkable GCAGA-containing sequence is conserved in the published genomes of *S. gallolyticus* UCN34, ATCC43143, and BAA-20690 and in all isolates of our collection expressing Pil1 pilus (data not shown). However, these strains differed in the number of GCAGA repeats and in the profile of Pil1 expression (Fig. S3).

### Mapping of the transcription start site of *pil1*

Determination of the transcription start sites of the *pil1* operon in *S. gallolyticus* strain UCN34 by primer extension analysis using a primer within pilA showed two bands (Fig. 2C), one positioned upstream the GCAGA repeats (position -390 bp from ATG_pilA_) and the other within the downstream stem-loop structure (position -130 bp from ATG_pilA_) (Fig. 2D). The first transcription start site is preceded by a canonical -35 (TTGTGT) and -10 Pribnow (AATAAT) boxes, suggesting that it represents the site of initiation of transcription, whereas the second initiation site does not (Fig. 2D). We therefore hypothesized that the second signal corresponded to the pause of reverse transcriptase during elongation at this secondary RNA structure or a processed mRNA. Consistently, fusions between various DNA fragments from this region and a *lac* *Z* reporter gene confirmed that only the canonical promoter displayed a detectable activity (Fig. S1). Overall these results indicate that *pil1* transcription starts 400 nucleotides upstream from the first gene *pilA*.

### *pil1* promoter region is necessary to the heterogeneous expression of pilus

**Figure 3:**
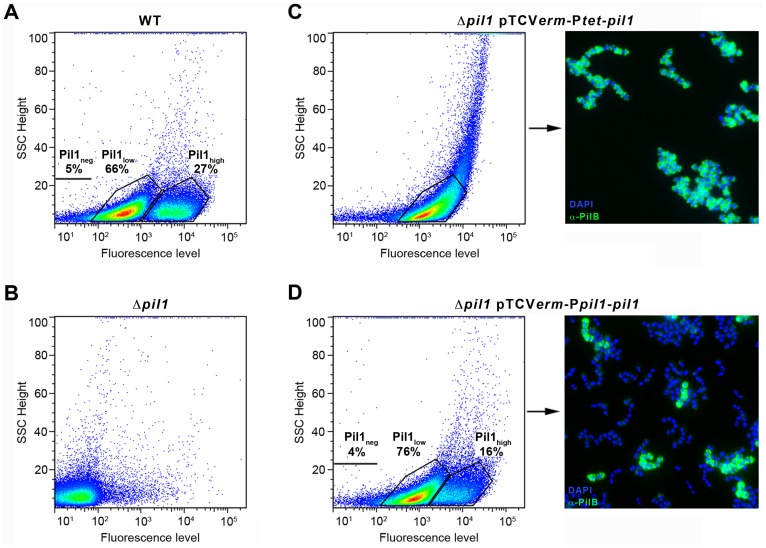
Heterogeneous expression of Pil1 depends on its 5′ upstream region. (A–D) Flow cytometry profiles of the WT UCN34 strain, the isogenic Δ*pil1* mutant, and the Δ*pil1* complemented with pTCV*erm*-P*tet*-*pil1* or pTCV*erm*-P*pil1*-*pil1*. (A) The WT strain displays two cell subpopulations, a majority of Pil1_low_ (66%) and a minority of Pil1_high_ (27%). (B) The Δ*pil1* mutant that does not express the Pil1 pilus. (C) The Δ*pil1* mutant complemented with the plasmid pTCV*erm*-P*tet*-*pil1*, P*tet* being a constitutive promoter, displays a single population of highly piliated cells. (D) The Δ*pil1* mutant complemented with the plasmid pTCV*erm*-P*pil1*-*pil1*, P*pil1* being the entire *gallo2180-pilA* intergenic region, displays two subpopulations. (C–D) Immunofluorescence staining with anti-PilB pAb (green) confirms that only the P*pil1* promoter and downstream sequences restored the heterogeneous expression of the Pil1 pilus (Pil1_low_, 76%; Pil1_high_, 16%). Bacteria were stained with DAPI (blue).

To determine if the heterogeneous expression of Pil1 pilus is controlled by the GCAGA-containing sequence, the *pil1* genes (*pilA, pilB* and *srtC*) were deleted in *S. gallolyticus* UCN34 (Δ*pil1*) and reintroduced in trans using two different expression plasmids. In plasmid pTCV*erm*-P*tet*-*pil1*, the *pil1* operon genes were transcribed from the constitutive promoter P*tet* fused immediately upstream the *pilA* gene, whereas in pTCV*erm*-P*pil1*-*pil1* it was transcribed from the UCN34 *pil1* promoter region, i.e. the 518-pb intergenic region located between *gallo2180* and *pilA* and containing the GCAGA repeats. Flow cytometry analyses with anti-PilB antibody showed that the Δ*pil1* mutant complemented with the plasmid pTCV*erm*-Ptet-*pil1* displayed a strong and homogeneous signal (Fig. 3C), whereas the complementation with pTCV*erm*-P*pil1*-*pil1* restored heterogeneity of *pil1* expression, with 76% of Pil1_low_ cells and 16% of Pil1_high_ cells (Fig. 3D). As expected, the Δ*pil1* mutant was negative for *pil1* expression (Fig. 1C and Fig. 3B). These observations were confirmed by immunofluorescence using anti-PilB antibody (Fig. 3C–D). Thus, this GCAGA-containing *pil1* promoter region controls the heterogeneous expression of *pil1* genes in *S. gallolyticus*.

### Isolation of UCN34 Pil1+ variants and phenotypic analyses

**Figure 4:**
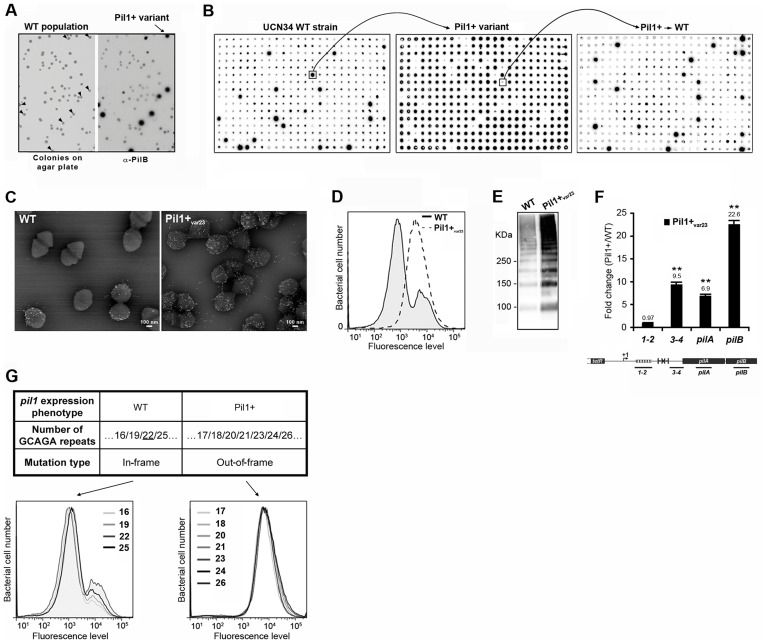
Isolation, phenotypic analyses and sequencing of Pil1+ variants. (A) Detection of Pil1+ variants by colony blot using anti-PilB pAb. Isolated bacteria on a TH agar plate (left) and corresponding replicate hybridized with anti-PilB pAb (right). Arrowheads indicate the few variants displaying a Pil1+ phenotype. (B) Serial analysis of Pil1 expression in WT and Pil1+_var_ populations. Individual colonies of WT UCN34 strain isolated on TH agar plates were grown in 96 well plates, transferred to a Nylon membrane, and probed with anti-PilB. Analysis of one Pil1+ variant revealed that it subsequently generates a majority of Pil1+ colonies (90–95%) and a minority of Pil1_low_ cells (5–10%) thus displaying phenotypic pilus heterogeneity as the WT UCN34. (C–F) Phenotypic comparison of WT UCN34 and Pil1+_var23_ variant. Scanning immunogold electron microscopy (C) and flow cytometry (D) analyses of bacteria labeled with anti-PilB pAb. Western blot analysis (E) of cell wall protein extracts revealed with anti-PilB pAb. Quantitative RT-PCR analysis (F) of RNAs extracted from exponentially growing *S. gallolyticus* cells. The expression levels were normalized using 16S rRNA as an internal standard and are indicated as the n-fold change with respect to the WT strain UCN34, expressed as means and standard deviations of at least three independent experiments with four technical replicates. Asterisks represent P values (*P<0.05) evaluated using a Student *t* test. The location in the *pil1* operon of the four primer pairs used is indicated at the bottom. (G) Sequence and flow cytometry analyses of WT UCN34 and Pil1+_var_ strains. Frameshift addition/deletion GCAGA repeats in the leader peptide encoding gene did not modify the Pil1 expression profile of UCN34 WT whereas out-of-frame addition were always associated with a Pil1+ phenotype.

To decipher the molecular mechanism underlying the heterogeneous expression of Pil1 pilus, UCN34 Pil1+ variants, highly enriched in Pil1_high_ cells, were separated from the wild type (WT) population by immunoscreening. Briefly, a colony-blot was performed on isolated colonies grown on TH plates with anti-PilB antibody. As shown in Fig. 4A, approximately 10% of the WT UCN34 colonies displayed a clear ‘Pil1+ phenotype’ with an approximately 10-fold increase of PilB level (referred from now as Pil1+_var_). Larger immunolabeling screening showed that 5 to 10% of clones with a Pil1+ phenotype were always obtained from UCN34 WT; similarly, 5 to 10% of the clones from a Pil1+ variant recovered the WT *pil1* expression level (Fig. 4B).

One variant named Pil1+_var23_ was further characterized phenotypically by immunogold electron microscopy, flow cytometry, Western blotting, and transcriptional analyses (Fig. 4C–F). As expected, Pil1+_var23_ displayed a high and homogeneous Pil1 expression profile. Interestingly, quantitative RT-PCR analyses indicated a strong increase of *pil1* operon transcription in Pil1+_var23_ compared to the WT UCN34 strain (Fig. 4F). However, as shown in Fig. 4F, this increase was only observed for transcripts located downstream from the putative stem-loop structure (Fig. 2E). Several other Pil1+ variants were analyzed and displayed similar phenotypic traits (data not shown). Of note, *pilB* transcripts are 3 to 4 fold more abundant than *pilA* specific transcripts (Fig. 4F). This might be due to differences in mRNA stability along the pilus operon and/or to additional regulatory mechanism controlling the level of *pilB* transcript.

### Sequencing of UCN34 Pil1+ variants

Direct sequencing of the *pil1* promoter region of UCN34 Pil1+ variants revealed modifications of the number of GCAGA repeats compared to the UCN34 WT strain, i.e. 21 or 23 repeats (respectively Pil1+_var21_ and Pil1+_var23_) instead of 22 repeats. Reversion from a Pil1+ to a WT phenotype was always accompanied by either a switch to the original 22 GCAGA repeats, or surprisingly acquisition of an in-frame number of repeats, i.e. 19 or 25. These WT-like populations displaying 19 or 25 repeats were able, in turn, to generate new *Pil1*+ variants displaying out-of-frame number (17, 18, 20, 21, 23, 24 or 26) of GCAGA repeats within the coding sequence of the putative peptide in the 5′ end of the transcript (Fig. 4G). From herein, we will refer to this peptide as leader peptide as defined by Molhoj and Degan [21]. In summary, WT-like expression of Pil1 pilus, with two distinct subpopulations of cells observed in flow cytometry (2/3 of Pil1_low_, 1/3 of Pil1_high_), was always correlated with alterations to a number of GCAGA repeats that was in-frame with the putative leader peptide (22+3n repeats). Otherwise, in the Pil1+ variants with an out-of-frame number or repeats (e.g. Pil1+_var23_), the population displayed a single homogeneous peak highly enriched in Pil1_high_ cells, as revealed by flow cytometry (Fig. 4G). This regulation of Pil1 expression due to addition or deletion of GCAGA repeats is reminiscent of a phase variation phenomenon.

### Increased transcription of *pil1* is translation-dependent

**Figure 5:**
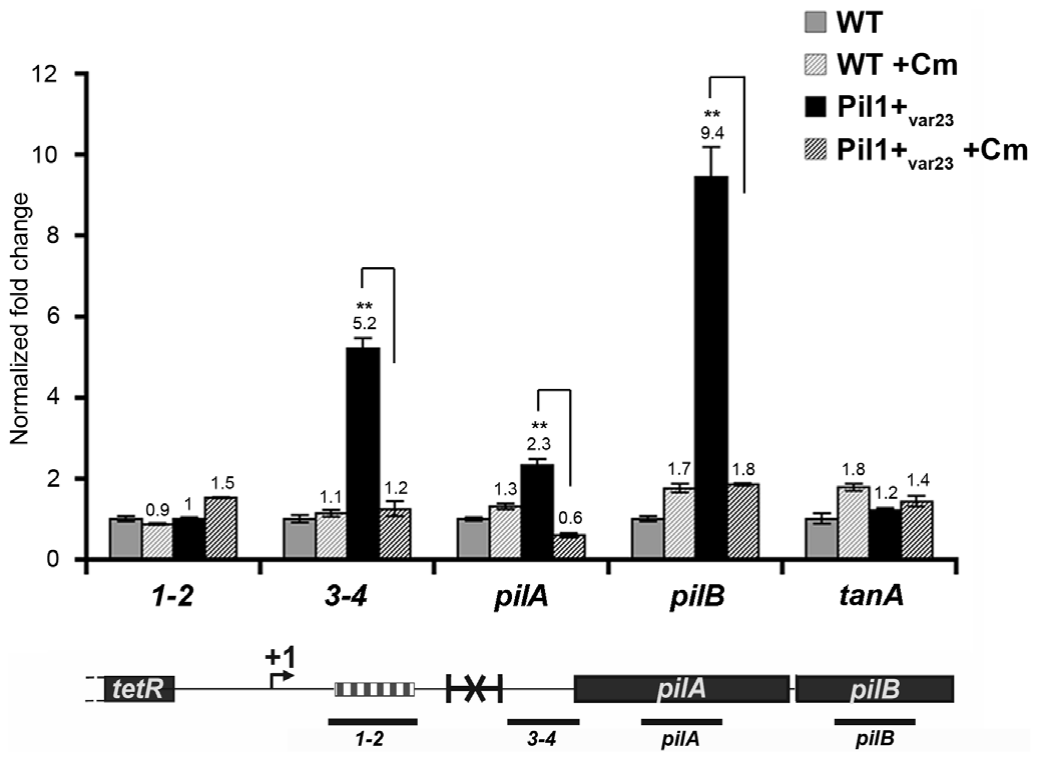
Uncoupling transcription and translation in the *pil1* operon. Quantitative RT-PCR were performed on RNAs extracted from UCN34 WT and Pil1+_var23_ variant grown without or with chloramphenicol (8 µg/mL) to induce ribosome stalling in leader peptide mRNA. The expression levels were normalized using 16S rRNA as an internal standard and are indicated as the n-fold change with respect to untreated WT strain UCN34, expressed as means and standard deviations of at least three separate experiments. The gene *tanA* was used as a reference gene whose transcription is not affected by addition of chloramphenicol. Asterisks represent P values (*P<0.05) evaluated using a Student *t* test. The location in the *pil1* operon of the four primer pairs used is indicated at the bottom.

The putative peptide encoded in the 518-bp *pil1* promoter region of Pil1+ variants with 21 or 23 in-frame GCAGA repeats terminates immediately upstream (4 bp) or within the hairpin structure, respectively. Strikingly, in UCN34 WT (22 repeats), this peptide ends at a stop codon located 49 bp upstream of the transcription terminator (Fig. 2D). We therefore hypothesized that translation of this putative leader peptide encoded by the region containing the GCAGA repeats was involved in *pil1* transcription. To test this hypothesis, we carried out quantitative RT-PCR analyses of the *pil1* operon genes with total RNA extracted from UCN34 WT strain and Pil1+_var23_ cultivated in the presence of chloramphenicol to uncouple transcription and translation. Addition of chloramphenicol to Pil1+ variants decreased the transcription of the *pil1* operon to a level similar to that observed in the WT strain (Fig. 5). Addition of chloramphenicol to UCN34 WT strain had no significant effect on the transcription of the *pil1* locus or of the control gene *tanA* (Fig. 5).

### Model of pilus regulation by an attenuation-like mechanism

**Figure 6:**
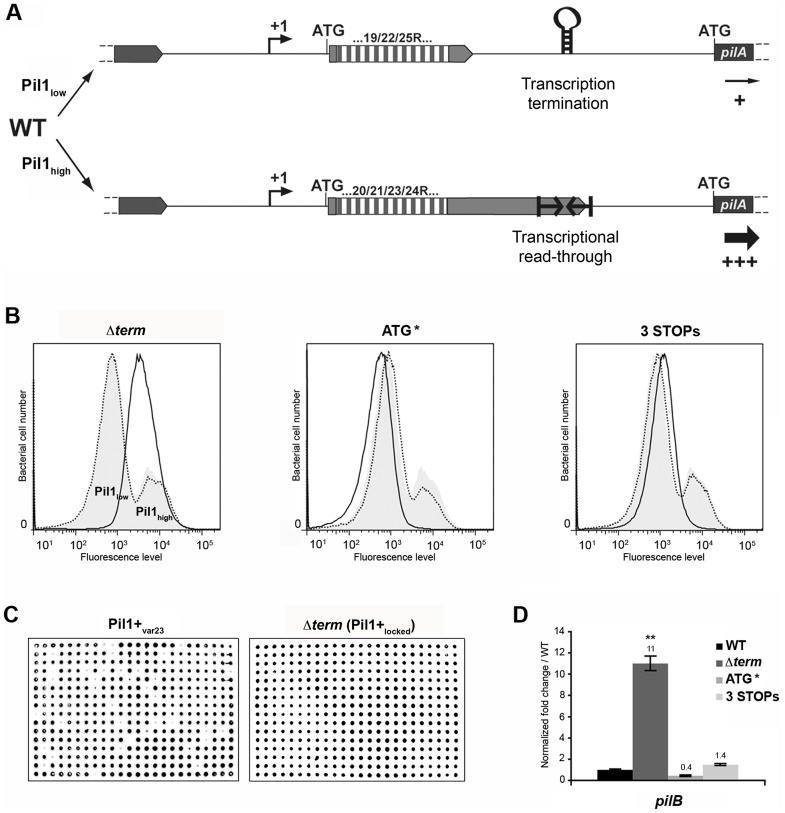
Model of pilus regulation by an attenuation-like mechanism. (A) *S. gallolyticus* UCN34 WT displays a heterogeneous *pil1* expression and consists of two main subpopulations, a majority of low piliated cells (Pil1_low_, 70%) and a minority of hyper piliated cells (Pil1_high_, 30%). The Pil1_low_ cells, characterized by a basal expression of *pil1*, possess a regulatory leader peptide-encoding gene with 22 GCAGA repeats that ends 39 bp upstream the hairpin transcription terminator. In-frame addition/deletion of GCAGA did not modify the distance between the leader peptide stop codon and the terminator. In this case, most transcripts initiated at P*pil1* promoter end at this premature terminator and the observed low expression of *pil1* probably occurs by readthrough transcription. The Pil1_high_ cells, characterized by a strong expression of *pil1*, displayed out-of-frame addition/deletion of repeats resulting in the synthesis of longer regulatory peptides whose stop codon are located within or dowstream of the hairpin terminator. Translation of these long regulatory leader peptides enhances *pil1* genes transcription by preventing the formation of the transcription terminator. (B) Flow cytometry analysis of UCN34 WT and isogenic mutant derivatives (Δ*term*, ATG*, and 3 STOPs) labeled with anti-PilB pAb. The WT and mutant profiles are depicted by gray area and black lines, respectively. Note that, as expected, the UCN34 and “back to the WT” profiles, depicted by dotted lines, are almost entirely superimposable. (C) Immunolabeling screening of the Pil1+_var23_ and Δ*term* strains with anti-PilB pAb. As opposed to the Pil1+_var23_ variant, the Δ*term* mutant is homogeneous and only generates Pil1+ colonies. This is the consequence of the deletion of the transcription terminator that blocks this strain in the Pil1_high_ configuration. (D) Quantitative RT-PCR were performed on RNAs extracted from WT UCN34 and mutant derivatives using pilB primers. The expression levels were normalized using 16S rRNA as an internal standard and are indicated as the n-fold change with respect to WT UCN34, expressed as means and standard deviations of at least three independent experiments with four technical replicates. Asterisks represent P values (** *P*<0.05) evaluated using a Student *t* test.

The above-described results led us to propose a model for the regulation of expression of pilus genes (Fig. 6A) where a regulatory leader peptide encoded in the 5′ end of the transcript and whose length varies upon phase variation (addition/deletion of GCAGA repeats), controls the switch of pilus transcription through translation-mediated anti-termination at the stem-loop structure acting as a premature transcription terminator upstream the *pil1* operon.

Flow cytometry analysis revealed that UCN34 WT population consists of two distinct subpopulations with 67% of the cells displaying low level of *Pil1* pilus (Pil1_low_) and 27% a high expression level of Pil1 (Pil1_high_) (Fig. 1B). According to our model, *Pil1*low cells should possess 22 GCAGA repeats leading to synthesis of a short leader peptide. Thus, most transcripts initiated at the promoter P*pil1* end at this hairpin structure, and the downstream *pil1* operon is transcribed at a low level (Pil1_low_ phenotype). In contrast, in Pil1_high_ cells, addition or deletion of a GCAGA repeat (e.g. 21 or 23, repeats) leads to a frameshift associated with synthesis of a longer leader peptide whose stop codon is located within or close to the transcription terminator. As the transcript-bound ribosome covers around 30 nucleotides it prevents the stem-loop formation and the downstream *pil1* operon is transcribed at a high level (Pil1_high_ phenotype). Thus, translation of this regulatory leader peptide at the 5′ end of the mRNA up-regulates *pil1* transcription by preventing the formation of the transcription terminator.

### Mutational analysis of *pil1* regulatory region

To further test the regulation model proposed in Fig. 6A, three mutations in the sequence encoding the putative leader peptide and the stem-loop structure were introduced in *S. gallolyticus* UCN34 chromosome: deletion of one strand of the stem-loop structure shown in Fig. 2E (Δ*term*); alterations of the RBS and ATG of the regulatory leader peptide (ATG* mutant where the GGAG of the RBS was replaced by CCTC and the ATG translational initiation codon by ACC); and addition of two stop codons at the end of the repeats (3 STOPs) to block translation in the three reading frames (Fig. S2). For each mutant generated in strain UCN34, we also selected a clone that reverted to the WT genotype (bWT) following homologous recombination. These bWT strains (standing for back to the WT) should display the WT phenotype and are isogenic to their mutant counterparts, i.e. they should possess the same secondary mutations, if any that may have occurred during their engineering. As shown in Fig. 6B, the Pil1 expression profile of each bWT strain was always superimposable to the WT UCN34. Flow cytometry analyses and immunolabelling were carried out to quantify the level of Pil1 using anti-PilB antibody (Fig. 6B, 6C and data not shown).

As predicted by our model, the *S. gallolyticus* UCN34Δ*term* mutant strain showed a high and homogeneous Pil1 expression by flow cytometry compared to the heterogeneous expression of the parental strain UCN34 (Fig. 6B). In addition, the immunoscreening of 384 individual clones clearly demonstrated that 100% of the cells in the Δ*term* population were trapped in the Pil1_high_ configuration and highly expressed pil1 (Pil1+_locked_ mutant); in contrast, 10% of the cells in the Pil1+ variants returned to a “WT *pil1* expression” by a phase variation mechanism (Fig. 6C). Quantitative RT-PCR analyses showed a 11-fold increased pilB transcription in the Δ*term* mutant compared to the parental UCN34 strain (Fig. 6D). These results are consistent with our proposal that the stem-loop structure upstream of the *pil1* genes acts as a premature transcription terminator. To further substantiate this finding, we have introduced the transcriptional terminator (TT) containing the run of 6T residues in the 3′ region downstream from the constitutive promoter P*tet* in the beta-galactosidase reporter vector pTCV-*lac* (annotated P*tet*-TT-*lac* *Z* ). As shown in Fig. S1, addition of the transcriptional terminator led to the reduction of *lac* *Z* reporter transcription (white/light blue colonies) compared to the control plasmid P*tet*-*lac* *Z* . Interestingly, as predicted for *E. coli* intrinsic terminators, deletion of the 6T residues in the 3′ region (annotated P*tet*-TTdelT-*lac* *Z* ) resulted in inactivation of the transcriptional terminator (strong blue colonies).

In the ATG* mutant, the ATG initiator codon of the regulatory leader peptide was replaced by ACC and its ribosome binding site drastically modified (GGAG to CCTC) (Fig. S2). Mutational inactivation of the leader peptide translation start signal led to a low and homogeneous *pil1* expression as shown by flow cytometry (Fig. 6B) and immunolabeling screening (not shown). Since the leader peptide is not translated, there is no opening of the transcription terminator and the heterogeneous expression is lost. As expected, the ATG* mutant displayed a significantly reduced amount of *pil1* transcription by quantitative RT-PCR compared to the parental strain UCN34. The remaining *pil1* expression, referred to as Pil1_low_, is probably due to a leak of the transcription terminator.

Finally, in the 3 STOPs mutant (Fig. S2) where only a short leader peptide was translated irrespective of the number of repeats (22, 18 or 10 repeats), a single peak was observed by flow cytometry (Fig. 6B, Fig. S6). Unexpectedly, the Pil1 expression level in these mutants is slightly higher than in the UCN34 WT Pil1_low_ cells subpopulation, a feature likely due to mutation-associated disturbance (e.g. increase mRNA stability).

Taken together, these results demonstrate that the translation of this regulatory leader peptide controls *pil1* expression.

### Functional significance of pilus heterogeneous expression

We previously showed that Pil1 was necessary and sufficient for binding to collagen [7]. Here, using isogenic mutants of *S. gallolyticus* UCN34, we showed a perfect correlation between Pil1 synthesis and the ability to bind to type I collagen (Fig. S5). These results confirmed that Pil1 is the major collagen-binding determinant of *S. gallolyticus*.

**Figure 7:**
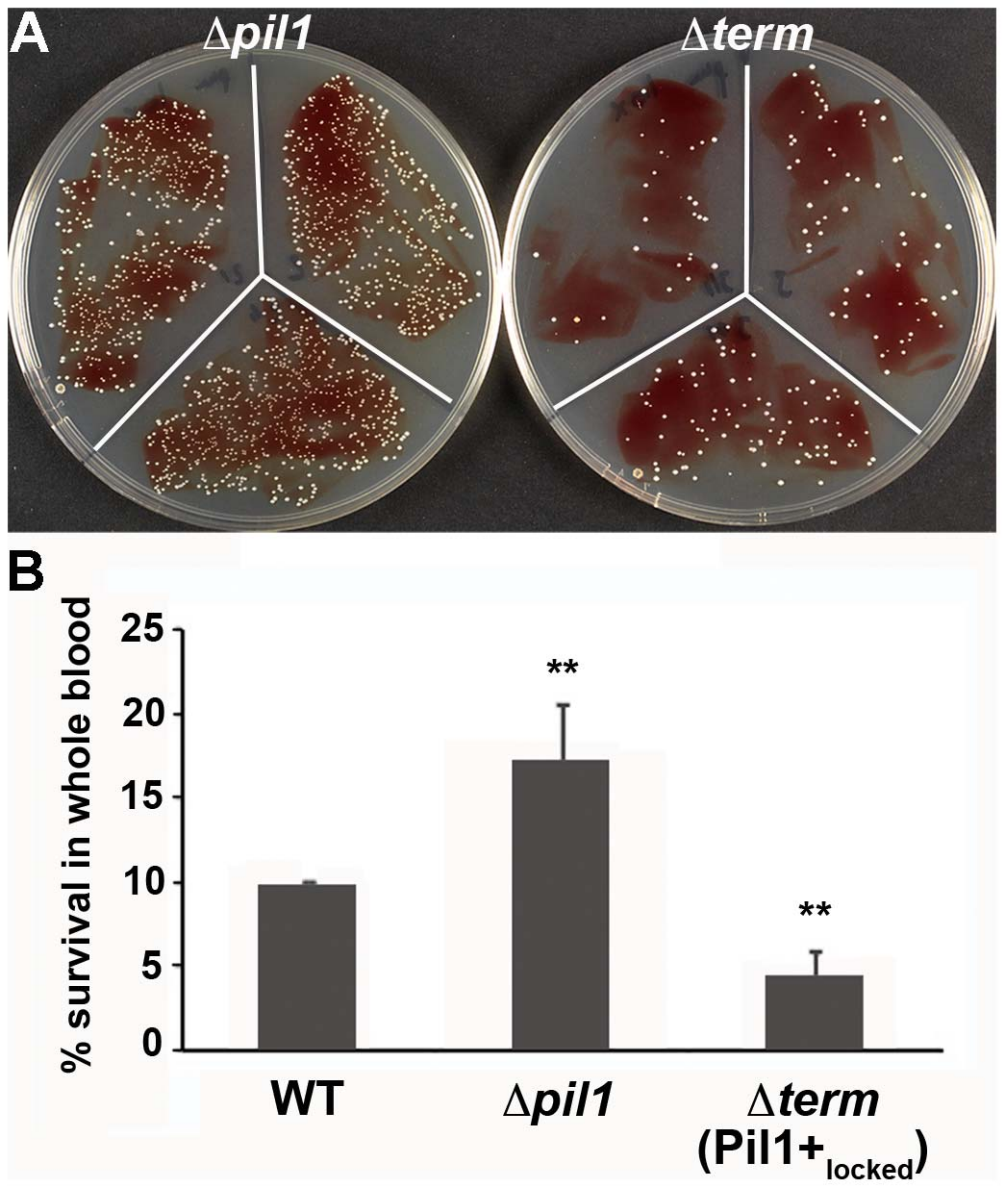
Whole blood survival of *S. gallolyticus* UCN34 WT, Δ*pil1*, and Δ*term* (Pil1+_locked_) strains. Exponentially growing bacteria were added to whole blood from three different human donors (10^4^ bacteria in 100 µl PBS were added to 400 µl of human blood drawn fresh in eppendorf tube, incubated on a wheel at 37°C and plated for enumeration at 1 h, 2 h and 3 h time points. (A) Plating on TH agar plates of Δ*pil1* and Δ*term* (Pil1+_locked_) strains after 3 h of incubation in whole blood at 37°C. The petri plate sectors represent three replicates of a bacterial survival assay with the blood of one donor at 2 h. (B) Percentage of whole blood survival for the three strains WT, Δ*pil1*, and Δ*term* (Pil1+_locked_) after 3 h of incubation. Values are represented as means +/− standard deviations of three independent experiments. Each experiment was performed in triplicate. Asterisks represent P values (** *P*<0. 05) evaluated using a Mann-Whitney test.

We previously showed that specific antibodies directed against *S. gallolyticus* pili, including Pil1, were present in the Dutch and American populations [22]. In addition, when raising polyclonal antibodies in rabbit and mice against the WT bacteria UCN34, a substantial proportion of the antibodies were directed against Pil1 pilins (data not shown). Therefore, although beneficial for colonization of host tissues, a high expression of Pil1 could be detrimental for evading the host immune response. To test experimentally the possible role of heterogeneous pilus expression in immune escape, we first assessed the survival of *S. gallolyticus* UCN34 WT, Δ*pil1* and Δ*term* (Pil1+_locked_) strains in human whole blood from three different donors. As shown in Fig. 7, the Pil1+_locked_ mutant was killed more efficiently than the Δ*pil1* mutant, most probably by neutrophils.

**Figure 8:**
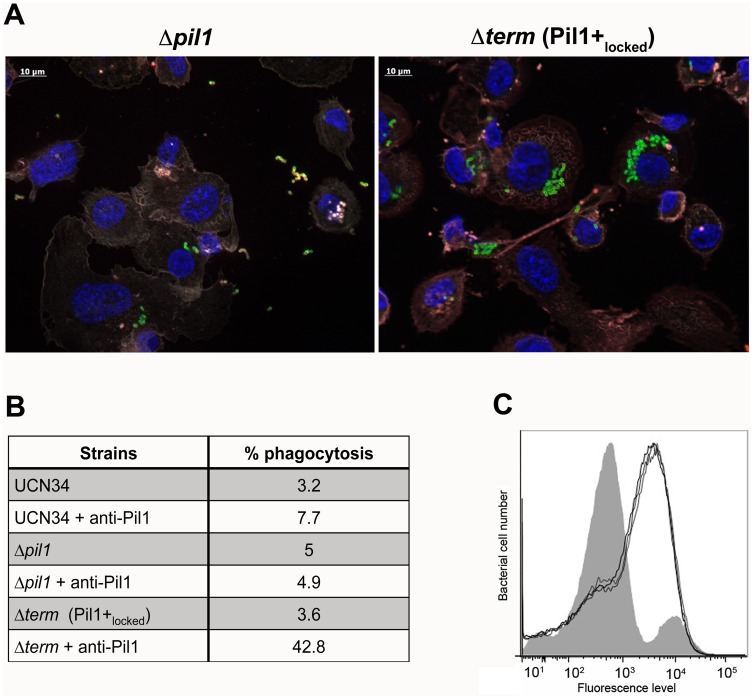
Uptake of *S. gallolyticus* UCN34 WT, Δ*pil1*, and Δ*term* (Pil1+_locked_) strains by human THP-1 macrophages. (A) Indirect immunofluorescence microscopy showing phagocytosis of Δ*pil1* or Δ*term* (Pil1+_locked_) mutants by THP-1 macrophages in the presence of anti-Pil1 antibody (opsonophagocytic assay). Actin is depicted in grey-purple, Hoeschst 33342 in blue, intracellular bacteria in green, and extracellular bacteria in yellow (green+red). (B) Percentage of phagocytosis by THP-1 human macrophages after 1 h of infection with WT, Δ*pil1*, and Δ*term* (Pil1+_locked_) strains, with or without an anti-Pil1 antibody. Values are representative of at least 4 independant experiments. (C) Flow-cytometry analysis of Pil1 expression performed on the UCN34 WT from the inoculum (grey) or from intracellular UCN34 bacteria recovered from THP-1 macrophages in an opsonophagocytic assay with an anti-PilB pAb (black lines are 3 replicates from one experiment).

We also infected the human monocyte-macrophage cell line THP-1 with UCN34 WT, Δ*pil1* and Δ*term* mutants in the presence or absence of purified antibodies against Pil1. *S. gallolyticus* UCN34, Δ*pil1* and Δ*term* (Pil1+_locked_) were poorly phagocytosed in the cell medium alone (≈2–5%). Addition of purified anti-Pil1 antibodies led to a 10-fold increase in the uptake of Pil1+_locked_ mutant via opsonophagocytosis but did not modify that of Δ*pil1* (Fig. 8A–B). Furthermore, flow-cytometry analysis of the intracellular UCN34 bacteria revealed that the bacteria phagocytosed by THP-1 macrophages in the presence of opsonizing antibodies corresponded mainly to the Pil1_high_ cells subpopulation (Fig. 8C).

## Discussion

Several recent publications have identified pili as playing a key role in the virulence of many gram-positive bacteria [17], [23]. Pili have been implicated in many facets of the infectious process such as adhesion/colonization of host tissues, translocation of epithelial barriers, and modulation of the innate immune responses. Their surface localization and high immunogenicity makes them attractive targets for the development of vaccines against important gram-positive pathogens such as Group B *Streptococcus* [24]. However, although the expression analysis of these important antigens is of obvious interest, very little is known about the regulation of pilus expression and how it is modulated by different environmental conditions.

Here, using single cell level analysis, we have shown that Pil1 pilus expression is heterogeneous in *S. gallolyticus* strain UCN34, with the co-existence of two subpopulations, a majority of weakly piliated (Pil1_low_) cells and a minority of hyper piliated (Pil1_high_) cells. We further demonstrated that this phenotypic variability depends on changes in the *pil1* promoter region encoding a leader peptide made of a variable number of repeats and a downstream transcription terminator. These two cis-acting elements control transcription of the downstream *pil1* genes. Sequence analysis of variants expressing high level of Pil1 pilus (referred to as Pil1+_var_), revealed out-of-frame addition/deletion of repeats compared to the parental UCN34 sequence; in contrast, clones displaying biphasic phenotype never exhibited sequence variation or in-frame addition/deletion of repeats. These results strongly suggested a phase variation mechanism involving simple sequence repeats (SSR) within the leader peptide-encoding gene. Accordingly, we postulate that translation of this leader peptide, whose length varies upon phase variation (addition/deletion of GCAGA repeats), controls the switch of pilus transcription through translation-mediated anti-termination at the stem-loop structure acting as a premature transcription terminator upstream the *pil1* genes (Fig. 6A).

The phenotypically distinct cell subpopulations of UCN34, Pil1_low_ and Pil1_high_, are present in variable ratios in other six *S. gallolyticus* isolates from our laboratory collection (Fig. S3). Sequencing of *pil1* promoter region of these strains confirmed the presence of GCAGA tandem repeats, but in different number. Similarly, the transcription terminator identified in UCN34 was found in all but one isolate (strain 2472) where another stem-loop structure is present at the same position. Isolates in which the regulatory leader peptide stops at least 28 nucleotides before the terminator (strains 2471, 2477, and 2479) displayed the same heterogeneous Pil1 expression profile as UCN34 (2/3 Pil1_low_ cells, 1/3 Pil1_high_ cells). In contrast, isolates in which the leader peptide terminates immediately upstream (strain 2475) or extends through the stem-loop structure (strains 2470 and 2472) displayed a high proportion of Pil1_high_ cells. Thus, the Pil1 expression profiles of these isolates could be predicted by our regulation model based on the sequence analysis of their *pil1* promoter regions. It is noteworthy that we could not find any external factors (temperature, pH, oxygen concentration, glucose, bicarbonate, serum, plasma, intestinal juice) modulating the two-third/one-third Pil1_low_/Pil1_high_ ratio in strain UCN34 (unpublished data). This result is consistent with a phase variation mechanism usually considered as a stochastic or random event and not as a responsive process.

We expressed *pil1* in *Lactococcus lactis* on a plasmid under the control of the promoter P*pil1* and we also observed heterogeneity in Pil1 expression, but the two populations were not as clearly separated as for *S. gallolyticus* (Fig. S7). In addition, we found a 23-repeat variant expressing *pil1* homogeneously at a high level. So it appears that the regulatory mechanism of *pil1* remains functional in this closely related species.

Sequence analysis revealed the presence of GCAGA repeats in the promoter region of another pilus locus (*pil3*) in the strain UCN34. Pil3 pilus expression is also heterogeneous, with two distinct subpopulations of cells observed by flow cytometry, Pil3_high_ and Pil3_low_ (Fig. S4). Strikingly, its promoter region also includes an ORF with 13 GCAGA tandem repeats encoding a putative regulatory leader peptide and a stem-loop transcription terminator (ΔG = −28.20 kcal/mol) located 33 bp downstream. Highly expressing Pil3+ variants all possessed out-of-frame addition of GCAGA repeats and encoded a longer regulatory leader peptide whose stop codon was located within or downstream of the hairpin structure. Similarly to Pil1, Pil3 expression is regulated by phase variation in *S. gallolyticus*.

Transcription attenuation is a commonly used regulation strategy characterized by the presence of an attenuator sequence involved in formation of mRNA stem-loops that prevent transcription of downstream genes. The best characterized transcription attenuation system is that of the *Escherichia coli* tryptophan (*trp*) operon [25]. At low intracellular tryptophan concentrations, the ribosome stalls during the translation of a tryptophan-containing leader peptide at a location that impedes the formation of a transcription terminator, thus enabling transcription of the *trp* operon. Attenuation of the *trp* operon is made possible by the fact that the rate of translation influences RNA structure, which in turn influences continuation of transcription. Translation therefore interferes with transcription, making this an example of translation-mediated transcription attenuation [26]. In the case of *pil1* operon, it is the length of the leader peptide varying upon addition/deletion of repeats that controls the transcription attenuation process.

Commonly described mechanisms of phase variation include gene inversion, gene conversion, epigenetic modifications and slipped-strand mispairing [27], [28]. Short sequence repeat (SSR) tracts, that undergo slipped-strand mispairing, are a major mechanism of stochastic switching of genes expression in bacterial commensals and pathogens such as *Haemophilus influen* *z* *ae, Escherichia coli, Salmonella enterica, Campylobacter jejuni,* *N* *eisseria gonorrhoeae* and *Helicobater pylori* [29]–[36]. In a SSR tract, a short unit of several nucleotides is repeated multiple times in the DNA sequence. During DNA replication, the process of *recA*-independent slipped-strand mispairing (or *recA*-dependent unequal crossing-over) can change the number of repeated units at a particular locus; this, in turn, can cause a shift in the reading frame, resulting in an out-of-frame translation, or alter critical spacing in the promoter region, which can, in both cases, “switch off” genes expression [27]. Mutations of this type are frequent and reversible, leading to rapid, stochastic *on-off* switching of expression of the gene and associated phenotype [27], [37]. Such populations may thus be “primed” for rapid adaptation [38]. SSRs have a high prevalence among surface associated proteins, such as pili and adhesins, probably due to their direct interactions with host structures and as targets of the immune responses [37]. In *S. gallolyticus* strain UCN34, the phase variation rate of the *pil1* locus, as defined by Eisenstein, is estimated to be 5.10^−3^/cell/generation [39], which is compatible with the rates (10^−3^ to 10^−4^/cell/generation) commonly reported, such as for *opa* genes in *N* *eisseria gonorrheae* [40]. This high mutation frequency is a characteristic of slipped-strand mispairing mechanism [27], [37].

Expression of highly immunogenic pili proteins involves a fitness cost due to the selective pressure of host immune responses. Hence, heterogeneous pili expression allows a subset of bacteria to be phenotypically pre-adapted to take advantage of particular environments and/or to avoid adverse conditions. The highly piliated subpopulation could allow colonization of specific host tissues exposing collagen, while the weakly expressing subpopulation is more prone to dissemination because it is less adherent to host tissues and may escape the host immune response. The hypothesis of a facilitated immune escape through phase variation has been demonstrated for major surface exposed structures such as LPS and PorA in *N* *eisseria meningitidis* [41], [42]. Consistently, we showed here that the Pil1+_locked_ mutant (Δ*term*) survived less in human whole blood than the Δ*pil1* mutant. This result indicates that neutrophils constituting the first line of innate host defenses better recognized bacteria that highly express Pil1, and killed them efficiently. We also showed that the non-piliated Δ*pil1* mutant better escape to opsonophagocytosis by THP-1 human macrophages than the Pil1+_locked_ mutant (Δ*term*). Furthermore, we have shown that among the heterogeneous Pil1 expressing WT bacteria, the bacteria that are phagocytosed by THP-1 are those expressing higher Pil1 levels i.e. the Pil1_high_ cells subpopulation.

This study describes the first example of pilus regulation through phase variation in a gram-positive pathogen. At the molecular level, it occurs through an original mechanism that combines phase variation in a leader peptide-encoding gene and transcription attenuation. Phase variation stands in contrast to other classical regulatory mechanisms, involving stand-alone or two-component regulators that tend to drive the entire bacterial population into an alternative expression status. This relatively simple yet robust regulation mechanism ensures a stochastic heterogeneous pilus expression at the bacterial surface, a feature important for evading the host immune system and to ensure optimal colonization versus dissemination in host tissues.

## Materials and Methods

### Ethics statement

Blood collection from human healthy volunteers was supplied by the ICAReB Plateform at the Institut Pasteur (Paris, France) in accordance to the guidelines of the agreement between Institut Pasteur and the Etablissement Français du Sang.

### Cell culture, bacterial strains, plasmids, and growth conditions

The human monocytic cell line THP-1 was cultured in RPMI-1640 GlutaMAX™ medium (Gibco reference 61870-010) supplemented with 10% FCS in a 10% CO2 atmosphere at 37°C. Phorbol 12-myristate 13-acetate (PMA) was used at 50 ng/mL to induce THP-1 monocytes to differentiate into macrophages.

Bacterial strains, plasmids and oligonucleotide primers are listed in Table S1. *S. gallolyticus* strains were grown at 37°C in Todd-Hewitt (TH) broth in standing filled flasks. *L. lactis* strain NZ9000 [43] was grown in M17 medium supplemented with 1% glucose (M17G). Heterologous expression of *pil1* in *L. lactis* strain was described previously [7]. Tetracycline and erythromycin were used at 10 µg/mL.

### Immunogold electron microscopy

For scanning electron microscopy (SEM), bacteria were collected after overnight growth, fixed, and stained with rabbit anti-PilB IgG followed by anti-rabbit secondary antibody conjugated to 10 nm colloidal gold as previously described [44].

### Immunofluorescence

*S. gallolyticus* or *L. lactis* recombinant strains were grown overnight in 10 ml of TH (OD600≈2) or M17G supplemented with erythromycin (OD_600_≈4) respectively. Bacteria were washed twice in phosphate buffered saline (PBS) before fixation in PBS containing 3% paraformaldehyde for 15 min at RT. Fixed bacteria were washed twice with PBS, blocked with PBS-BSA 3% for 30 min, and incubated for 45 min with rabbit or mouse primary antibodies diluted in PBS-BSA 0.5% at the following dilutions: anti-PilB (1/800) and anti-PilA (1/500). After three washings with PBS, samples were incubated for 30 min with secondary DyLight 488-conjugated goat anti-rabbit or mice immunoglobulin diluted in PBS-BSA 0.5% (1/300 dilution; Thermo Scientific Pierce) and Hoecht 33342 (1/1,000). Coverslips were mounted with 4 µl of Fluoromount-G mounting medium (SouthernBiotech). Microscopic observations were done with a Nikon Eclipse Ni-U and images acquired with a Nikon Digital Camera DS-U3.

### Flow cytometry analysis

To analyze Pil1 pilus expression by flow cytometry, 500 µl of overnight cultures were collected and washed twice in PBS. Bacterial pellet was resuspended in PBS-BSA 3% blocking solution for 30 min, and then incubated with rabbit anti-PilB (1/800) or anti-PilA (1/500) antisera diluted in PBS-BSA 0.5% for 45 min on ice. After three washes with PBS, samples were incubated with secondary DyLight 488-conjugated goat anti-rabbit or anti-mouse immunoglobulin (Thermo Scientific Pierce) diluted in PBS-BSA 0.5% for 30 min on ice. Cells were washed before fixation in PBS containing 1% paraformaldehyde for 20 min. Samples were acquired on a MACSQuant Analyzer apparatus (Miltenyi Biotec) and data were analyzed using FlowJo software.

### Primer extension reactions

Total RNA was used as template for primer extension reaction using a radiolabeled specific primer complementary to a sequence located downstream the putative *pil1* promoter region, CD37 (Table S1), as previously described [45]. The corresponding Sanger DNA sequencing reactions (GATC) were carried out by using the same primer and a PCR-amplified fragment containing the *pil1* upstream region (primer pair CD35/CD37) with the Sequenase PCR product sequencing kit (USB).

### Beta-galactosidase assay

Transcriptional fusions of different promoter regions with a *spoVG-lac* *Z* reporter gene have been performed using pTCV*lac* using *Eco*RI/*Bam*HI restriction sites, and the various plasmids were introduced into *Streptococcus agalactiae* NEM316. Overnight cultures were diluted 1 in 100 in 10 ml of fresh TH broth with erythromycin and grown for 4–5 h at 37°C. The β-galactosidase activities were measured using the Beta-Glo assay system (Promega, WI) according to the manufacturer's recommendations on exponentially growing bacteria (OD_600_nm = 0.7). The assay consisted of mixing an equal volume (50 µl) of a bacterial suspension in PBS (adjusted to OD_600_nm of 1) with the Beta-Glo Reagent containing a luciferin-galactoside substrate (6-O-β-galactopyranosyl-luciferin). After 1 h incubation in the dark, the light produced was measured in a luminometer Lumat LB 9507 tube (Berthold Technologies). The results are expressed as relative light unit (RLU) per OD_600_nm, and are representative of at least three independent experiments.

### Preparation of cell wall protein extracts and immunoblotting

Bacteria were grown in TH medium at 37°C and harvested for protein analysis during late exponential phase of culture. Cell wall extracts were prepared as previously described (24). For analysis of PilB expression by Western immunoblotting, cell wall proteins were boiled in Laemmli sample buffer, separated by SDS-PAGE on 4–12% Tris-Acetate Criterion XT gradient gels and transferred to nitrocellulose membrane (Hybond-C, Amersham). PilB was detected using specific polyclonal antibodies and horseradish peroxidase (HRP)-coupled anti-rabbit secondary antibodies (Zymed) and the Western pico chemiluminescence kit (Pierce). Image capture and analysis were done on GeneGnome imaging system (Syngene).

For colony blots, 100 µl of a 10-6 dilution of overnight culture was spread on TH agar plates, incubated for 24 h at 37°C and transferred onto nitrocellulose (Hybond-C, Amersham). The membrane was dried in the microwave (3 min at 300 W) and soaked in blocking solution of PBS with 3% skimmed milk for 30 min. Immuno-detection was performed as described above.

For immunolabeling screening, 384 isolated colonies of each strain grown on agar plates were inoculated in 100 µl of TH medium in four 96-well plates. After 8 h culture at 37°C, the 96-well plates were gently shaken for 15 s and each plate was replicated on TH agar plates with a robot Rotor HDA (Singer Instruments). Following overnight incubation at 37°C, the four plates were combined on a single TH agar plate containing 384 clones. This master plate was duplicated and transferred onto nitrocellulose membrane (Hybond-C, Amersham) for immuno-detection.

### Sequencing

For sequencing of short regions, genomic DNA was prepared with the DNeasy Blood & Tissue kit (Qiagen), and sequencing reactions were performed on 10 µl of DNA matrix with the BigDye Terminator v3.1 Cycle Sequencing kit (Applied Biosystems). Genomic DNA of variants (Pil1+_var23_; 23 GCAGA repeats) and mutant strains (Δterm, ATG*) were extracted using MasterPure Gram Positive DNA Purification Kit (Epicentre, Illumina) and genomes were sequenced by Next-Generation Sequencing (NGS) technique to confirm the absence of disturbing secondary mutations. The samples were sent to the Institut Pasteur platform PF1 for sequencing.

### Quantitative Real-time Reverse Transcriptase-Polymerase Chain Reaction (RT-PCR)

Total RNA (15 µg) were extracted and treated as described previously [46]. Quantitative RT-PCR analyses were performed as previously described [46] with gene-specific primers (Table S1). For treatment with chloramphenicol, the antibiotic was added to 20 ml of culture in exponential phase (OD_600_nm = 0.3) to 8 µg/mL, a concentration that inhibits bacterial growth. The mock control consists of the same volume of culture with the diluent (ethanol). After 30 min at 37°C, the cultures were harvested for RNA extraction.

### Mutant construction

Experimental details concerning the construction of the *S. gallolyticus* UCN34 Δ*pil1* mutant deleted for the three genes constituting the *pil1* operon (*gallo2179-2178-2177*) is the subject of a manuscript describing a method to construct isogenic mutants in this non-naturally transformable species (Danne et al., in preparation). The same technique has been used to construct Δ*term*, ATG* and 3 STOPs mutants (for primers, see Table S1).

### Phagocytosis assay

Human monocytic THP-1 cells were seeded into 24-well plates at 5. 10^5^ cells per well in RPMI-1640 GlutaMAX supplemented with 10% FCS and PMA (50 ng/mL) and incubated for 48 h prior to phagocytosis. This assay was performed on exponentially grown bacteria (OD≈0.4) washed once in PBS and diluted in RPMI-1640 GlutaMAX medium. When indicated, purified antibodies against the major pilin (Gallo2178 or PilB) were added to the bacteria (dilution 1/200) for 15 min at room temperature before addition to the host cells. Macrophages were infected at a multiplicity of infection (m.o.i.) of 10 bacteria per cell and incubated for 1 h to allow phagocytosis. Extracellular bacteria were killed with 50 µg/mL gentamicin for 1 h at 37°C. Viable intracellular bacteria (cfu) were determined by macrophage lysis in 1 ml of cold water. The percentage of phagocytosis was calculated as follows (cfu on plate count/cfu in original inoculum ×100). Assays were performed in triplicate and were repeated at least three times. Similar experiments were performed on coverslips and used for indirect immunofluorescence microscopy. After phagocytosis, the infected THP-1 cells were fixed in PBS containing 4% paraformaldehyde for 15 min at room temperature. Non-specific binding sites were blocked with PBS BSA 3% for 30 min at room temperature. Extracellular bacteria were labeled using a rabbit polyclonal antibody directed against *S. gallolyticus* UCN34 and revealed with AlexaFluor 594-conjugated goat anti-rabbit immunoglobulin (1/200 dilution in PBS-BSA 1%, Molecular Probes, Invitrogen). Next, the macrophages were permeabilized with PBS-Triton X-100 (0.2%) for 5 min at room temperature. Bacteria were labeled using a rabbit polyclonal antibody directed against *S. gallolyticus* UCN34 and revealed with AlexaFluor 488- conjugated goat anti-rabbit immunoglobulin (1/200 dilution in PBS-BSA 1%, Molecular Probes, Invitrogen). The THP-1 macrophages were labeled with Cy3-conjugated phalloidin (1/300 dilution) to visualize the actin network and with Hoeschst 33342 (10 µg/mL) to detect the nuclei. Using this differential staining technique, intracellular bacteria will appear only labeled in green whereas extracellular bacteria will appear both in green and red ( = yellow). Images are acquired with Apotome Zeiss and Axiovision 4.6 software. This system provides an optical slice view reconstructed from fluorescent samples, using a series of “grid projection” (or “structured illumination”) acquisitions.

## Supporting Information

Figure S1 **Transcriptional** ***lacZ*** **fusions to map mRNA start sites in the** ***pil1*** **promoter.** Transcriptional fusions of different promoter regions with the *lac* *Z* reporter gene pTCV*lac*. (A) Luminescence measurements (RLU) were performed on exponentially growing *Streptococcus agalactiae* NEM316 cells using the Beta-Glo Assay System (Promega). P-: no promoter (negative control); P*cyl*: strong constitutive promoter (positive control); P*aphA-3*: weak constitutive promoter (positive control); P*pil1*: region identified by primer extension (see the band designated as “+1” in Fig. 2C) and containing a putative promoter sequence and the 22 GCAGA repeats; “*”: region identified by primer extension (see the band designated as “*” in Fig. 2C) lying within a putative terminator. The arrows indicate the positions of the putative transcriptional start sites as determined by primer extension. (B) Beta-galactosidase activity on TH agar plate containing X-gal substrate. P*tet*-*lacZ* is the control plasmid in which the *lac* *Z* gene is under the control of the constitutive promoter P*tet*. P*tet*-TT-*lac* *Z* contains the entire pil1 transcriptional terminator (TT) with the 6T residues in its 3′ region. P*tet*-TTdelT-*lac* *Z* is identical to P*tet*-TT-*lac* *Z* except for the removal of the 6T residues in the 3′ region of the transcriptional terminator.
(TIF)

Figure S2 **Schematic representation of the targeted mutants in the 5′ region of** ***pil1*** **.** Nucleotide sequence of the mutated *pil1* re ulatory regions (Δ*term*, ATG* and 3 STOPs). The GCAGA repeats are in bold characters, the stem-loop structure is underlined, and the mutated nucleotides are indicated in gray.
(TIF)

Figure S3 **Pil1 pilus heterogeneity in** ***S. gallolyticus*** **isolates.** Nucleotide sequence of the intergenic *gallo2180-pilA* region in six clinical isolates of *S. gallolyticus* named 2470, 2471, 2472, 2475, 2477, 2479. The sequence encoding the leader peptide is in bold, the main and alternative STOP codons, due to phase variation, are shaded and the stem-loop structure is underlined. The number of GCAGA repeats, the distance between the leader peptide STOP codon and the first nucleotide of the transcription terminator are indicated. The corresponding Pil1 profiles are shown on the right.
(TIF)

Figure S4 **Heterogeneity of Pil3 pilus.** Two subpopulations expressing low level (Pil3_low_) and high level (Pil3_high_) of Pil3 pilus are present in the UCN34 population, as revealed by flow cytometry using a specific Pil3 antibody (i.e. the major pilin Gallo2039) and the DyLight 488 rabbit secondary antibody. The two peaks in black represent Pil3_low_ (70%) and Pil3_high_ (28%) cells subpopulation in strain UCN34. The profile of a Pil3+ variant containing 15 GCAGA repeats (Pil3+_var15_) is shown in gray.
(TIF)

Figure S5 **Binding of** ***S. gallolyticus*** **isogenic mutants to collagen.** (A) Flow cytometry analysis of *S. gallolyticus* UCN34 WT, Δ*pil1*, Pil1+_var23_, Δ*term* (Pil1+_locked_), ATG* and 3 STOPs strains labeled with anti-PilB pAb. The WT profile is depicted by a gray area, the different mutants by solid colored lines and the Pil1+ variant by a dashed red line. (B) Adherence of *S. gallolyticus* UCN34 WT, Δ*pil1*, Pil1+_var23_, Δ*term* (Pil1+_locked_), ATG* and 3 STOPs strains binding to immobilized collagen type I and fibrinogen (loading control). Microtiter wells were coated with 10 µg of ECM proteins, and 10^7^ bacterial colony-forming units were added. The wells were washed, and bound bacteria were detected using crystal violet (CV) staining. Optical density at 595 nm (OD_595 nm_) values are presented as means +/− standard deviations for 3 experiments performed in triplicate. Abbreviations: Col I, collagen I; Fg, fibrinogen. Asterisks represent P values (** *P*<0.01, * *P*<0.05) evaluated using a Mann-Whitney test.
(TIF)

Figure S6 **Flow cytometry analysis of UCN34 WT and 3 STOPS mutants with 22R, 18R and 10R GCAGA repeats.** All mutants displayed a homogeneous profile when labeled with anti-PilB pAb as compared to the WT profile depicted in grey.
(TIF)

Figure S7 **Heterogeneous expression of Pil1 in** ***Lactococcus lactis*** **NZ9000 depends on its 5′ upstream region.** Flow cytometry profiles of *L. lactis* NZ9000 strains expressing pTCVerm, pTCV*erm*-P*tet*-*pil1*, or pTCV*erm*-P*pil1*-*pil1* containing 22 or 23 GCAGA. Note that *L. lactis* NZ9000 pTCV*erm*-P*pil1*-*pil1* with 22 repeats displays a heterogeneous expression of Pil1 with two subpopulations whereas a single population of highly piliated strains was observed with pTCV*erm*-P*pil1*-*pil1* containing 23 repeats. As expected, *L. lactis* cells where *pil1* is transcribed from the constitutive promoter P*tet* (pTCV*erm*-P*tet*-*pil1*,) display a single population of highly piliated cells whereas *L. lactis* expressing the empty vector pTCV*erm* do not express Pil1.
(TIF)

Table S1 **Bacterial strains, plasmids and primers.**
(DOC)
