# Supplementary figures and images for: Single Cell Stochastic Regulation of Pilus Phase Variation by an Attenuation-like Mechanism
Source: PLoS Pathog. 2014 Jan 16;10(1):e1003860. doi: 10.1371/journal.ppat.1003860 (PMC3894217; doi:10.1371/journal.ppat.1003860)

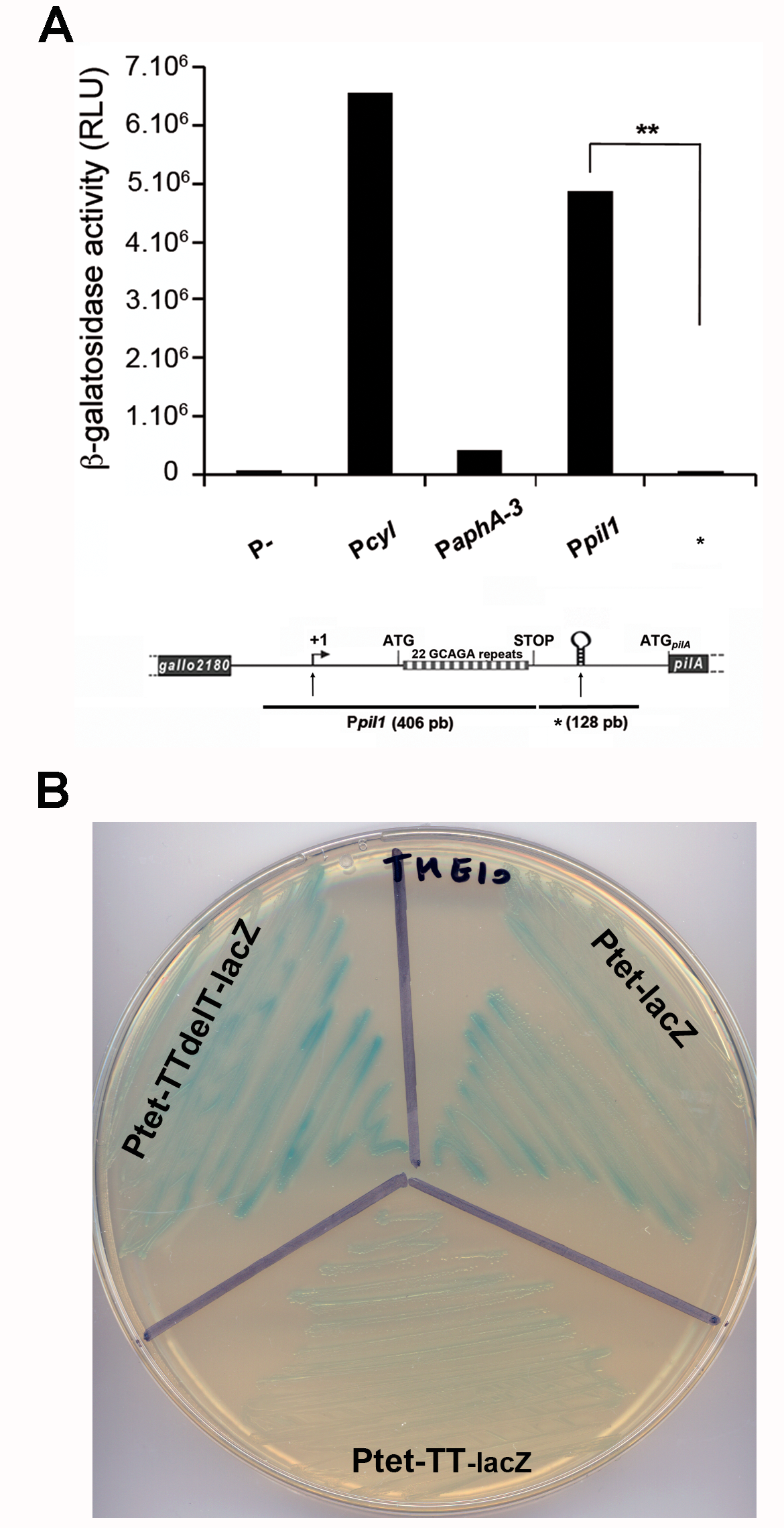

Supplement: Figure S1 — Transcriptional lacZ fusions to map mRNA start sites in the pil1 promoter. Transcriptional fusions of different promoter regions with the lac Z reporter gene pTCVlac. (A) Luminescence measurements (RLU) were performed on exponentially growing Streptococcus agalactiae NEM316 cells using the Beta-Glo Assay System (Promega). P-: no promoter (negative control); Pcyl: strong constitutive promoter (positive control); PaphA-3: weak constitutive promoter (positive control); Ppil1: region identified by primer extension (see the band designated as “+1” in Fig. 2C) and containing a putative promoter sequence and the 22 GCAGA repeats; “*”: region identified by primer extension (see the band designated as “*” in Fig. 2C) lying within a putative terminator. The arrows indicate the positions of the putative transcriptional start sites as determined by primer extension. (B) Beta-galactosidase activity on TH agar plate containing X-gal substrate. Ptet-lacZ is the control plasmid in which the lac Z gene is under the control of the constitutive promoter Ptet. Ptet-TT-lac Z contains the entire pil1 transcriptional terminator (TT) with the 6T residues in its 3′ region. Ptet-TTdelT-lac Z is identical to Ptet-TT-lac Z except for the removal of the 6T residues in the 3′ region of the transcriptional terminator. (TIF) [file ppat.1003860.s001.tif]

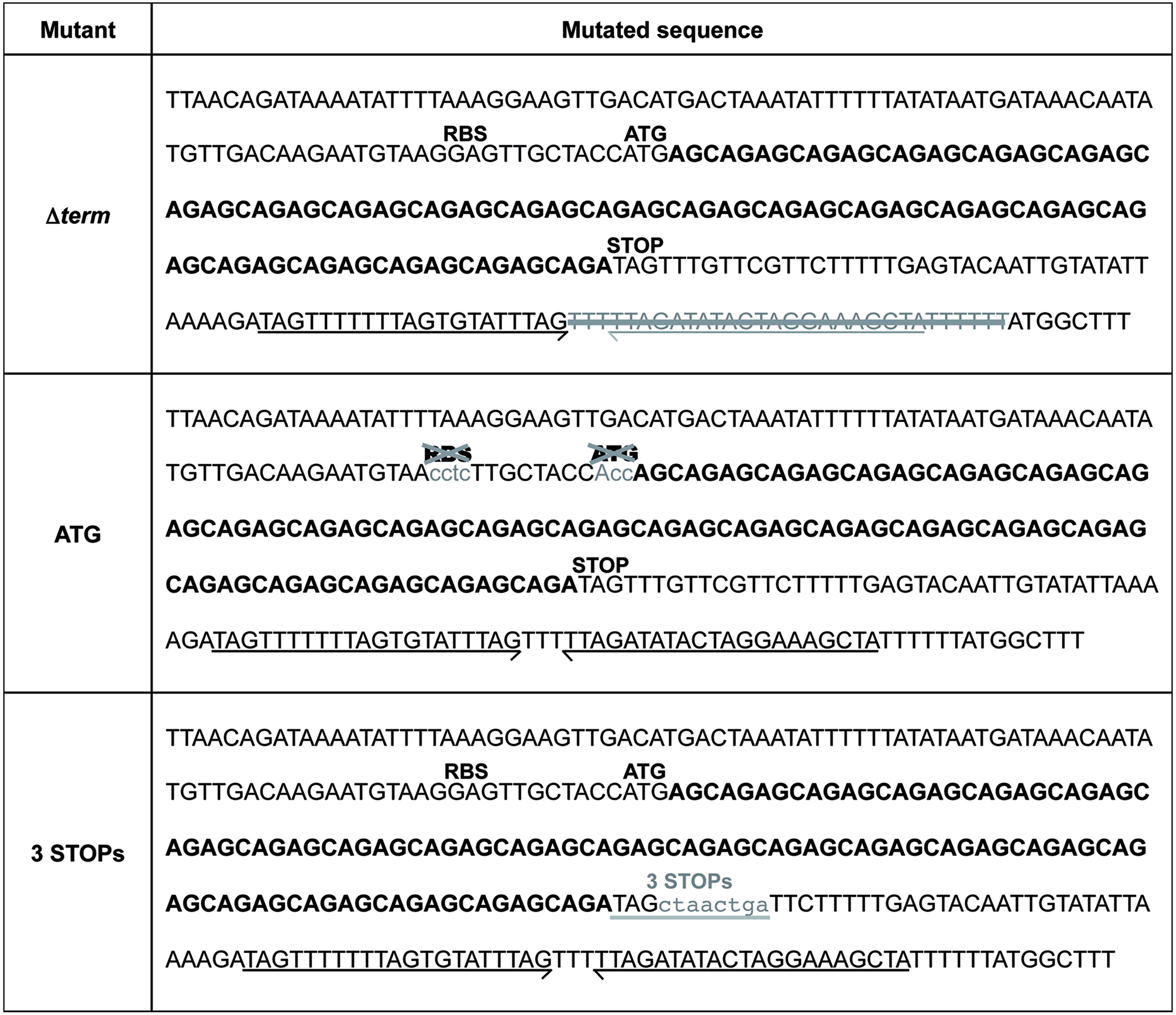

Supplement: Figure S2 — Schematic representation of the targeted mutants in the 5′ region of pil1 . Nucleotide sequence of the mutated pil1 re ulatory regions (Δterm, ATG* and 3 STOPs). The GCAGA repeats are in bold characters, the stem-loop structure is underlined, and the mutated nucleotides are indicated in gray. (TIF) [file ppat.1003860.s002.tif]

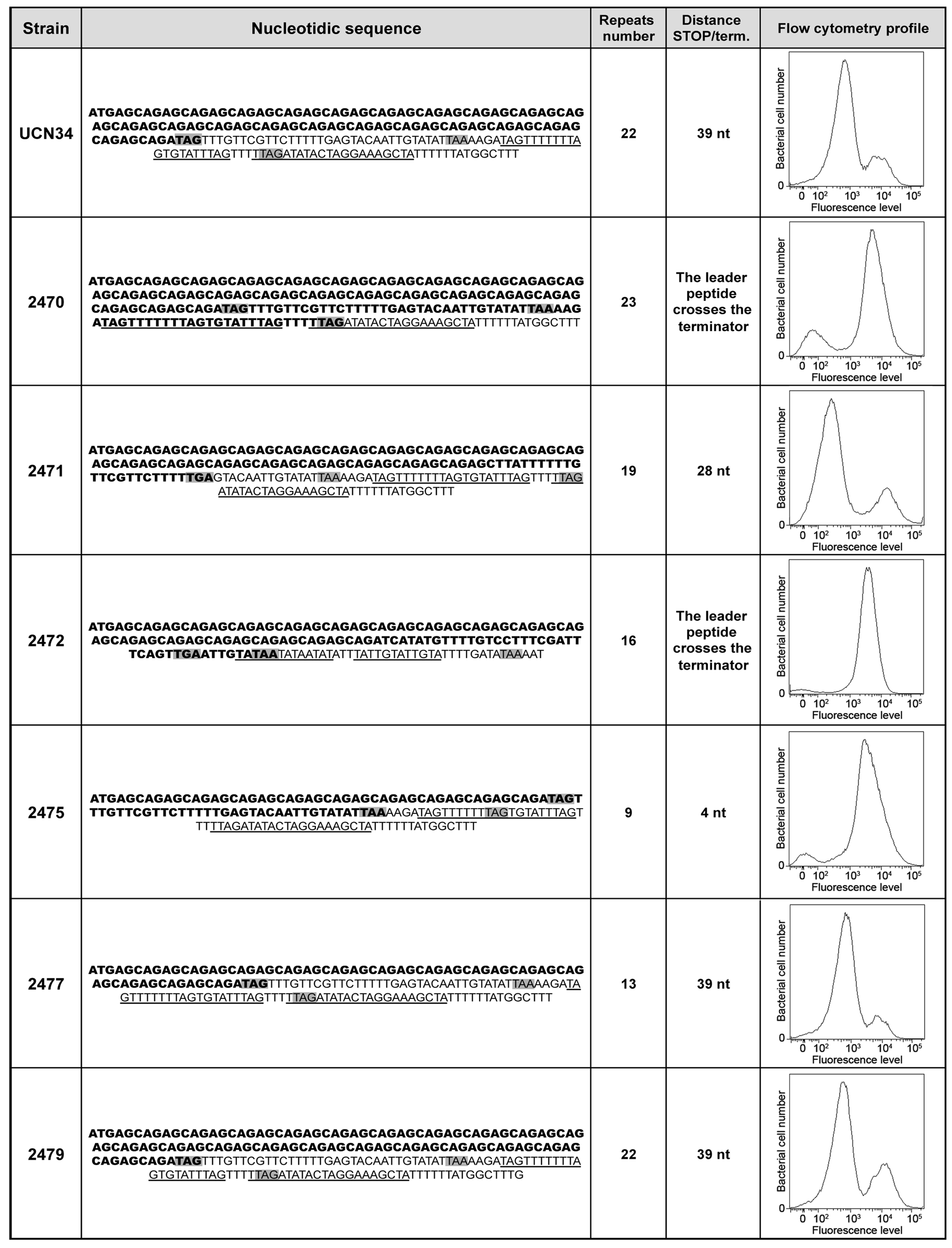

Supplement: Figure S3 — Pil1 pilus heterogeneity in S. gallolyticus isolates. Nucleotide sequence of the intergenic gallo2180-pilA region in six clinical isolates of S. gallolyticus named 2470, 2471, 2472, 2475, 2477, 2479. The sequence encoding the leader peptide is in bold, the main and alternative STOP codons, due to phase variation, are shaded and the stem-loop structure is underlined. The number of GCAGA repeats, the distance between the leader peptide STOP codon and the first nucleotide of the transcription terminator are indicated. The corresponding Pil1 profiles are shown on the right. (TIF) [file ppat.1003860.s003.tif]

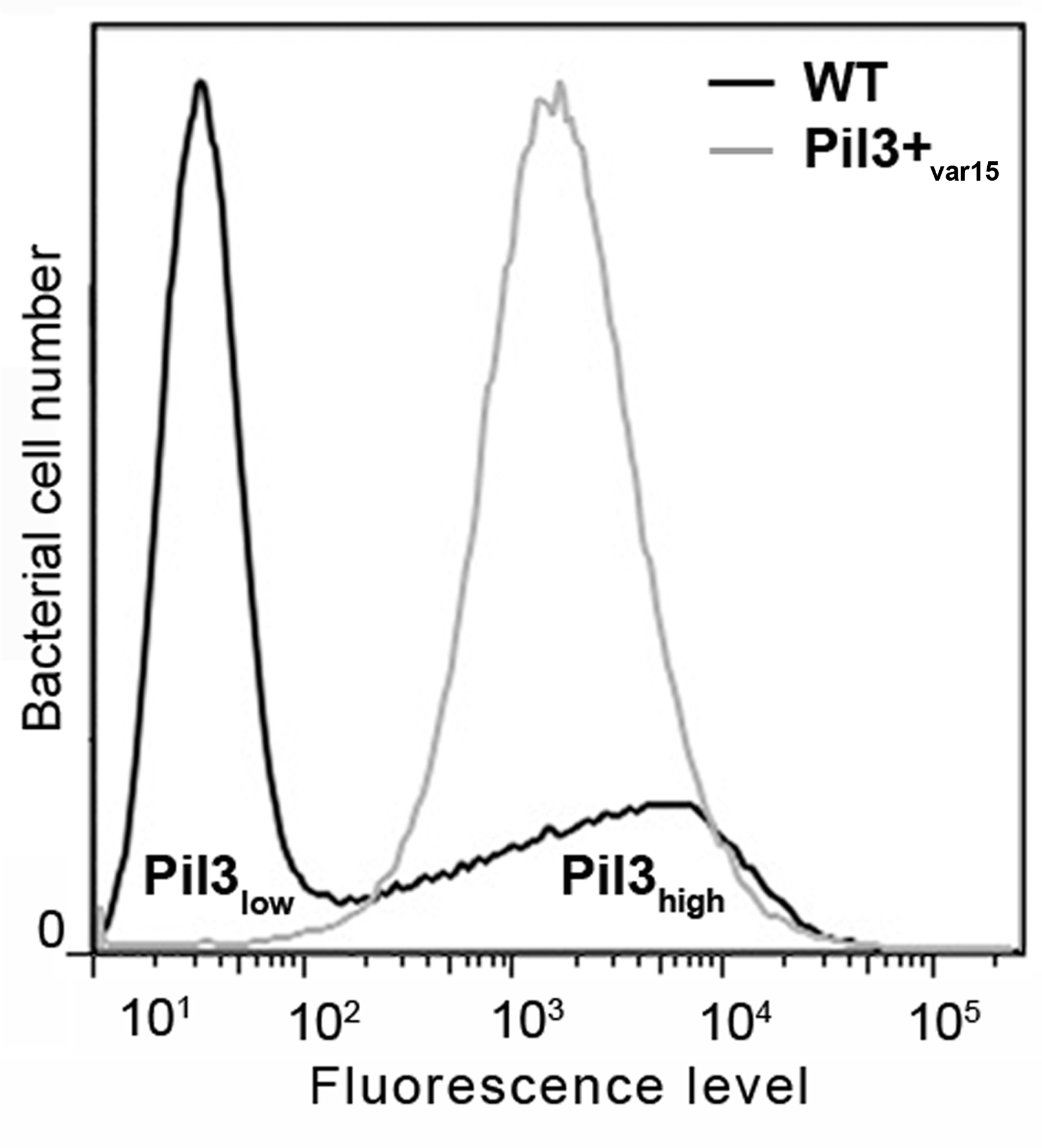

Supplement: Figure S4 — Heterogeneity of Pil3 pilus. Two subpopulations expressing low level (Pil3low) and high level (Pil3high) of Pil3 pilus are present in the UCN34 population, as revealed by flow cytometry using a specific Pil3 antibody (i.e. the major pilin Gallo2039) and the DyLight 488 rabbit secondary antibody. The two peaks in black represent Pil3low (70%) and Pil3high (28%) cells subpopulation in strain UCN34. The profile of a Pil3+ variant containing 15 GCAGA repeats (Pil3+var15) is shown in gray. (TIF) [file ppat.1003860.s004.tif]

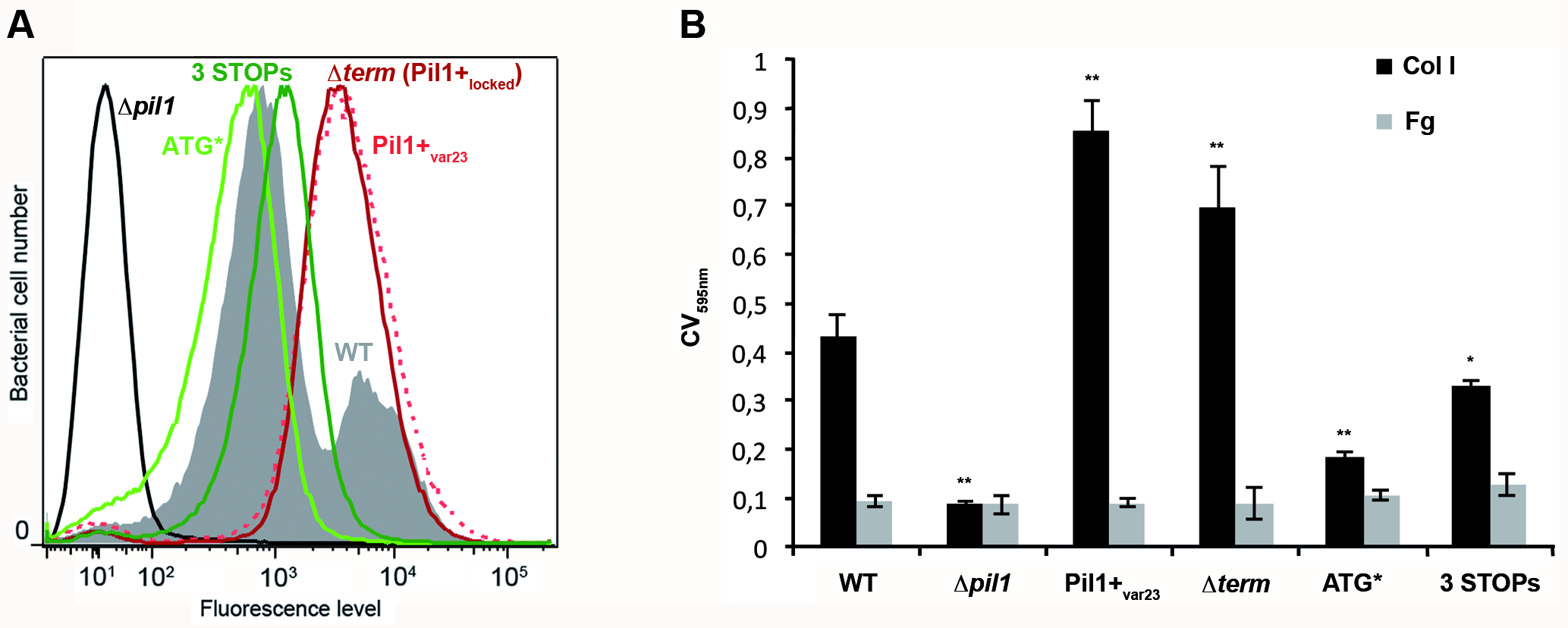

Supplement: Figure S5 — Binding of S. gallolyticus isogenic mutants to collagen. (A) Flow cytometry analysis of S. gallolyticus UCN34 WT, Δpil1, Pil1+var23, Δterm (Pil1+locked), ATG* and 3 STOPs strains labeled with anti-PilB pAb. The WT profile is depicted by a gray area, the different mutants by solid colored lines and the Pil1+ variant by a dashed red line. (B) Adherence of S. gallolyticus UCN34 WT, Δpil1, Pil1+var23, Δterm (Pil1+locked), ATG* and 3 STOPs strains binding to immobilized collagen type I and fibrinogen (loading control). Microtiter wells were coated with 10 µg of ECM proteins, and 107 bacterial colony-forming units were added. The wells were washed, and bound bacteria were detected using crystal violet (CV) staining. Optical density at 595 nm (OD595 nm) values are presented as means +/− standard deviations for 3 experiments performed in triplicate. Abbreviations: Col I, collagen I; Fg, fibrinogen. Asterisks represent P values (** P<0.01, * P<0.05) evaluated using a Mann-Whitney test. (TIF) [file ppat.1003860.s005.tif]

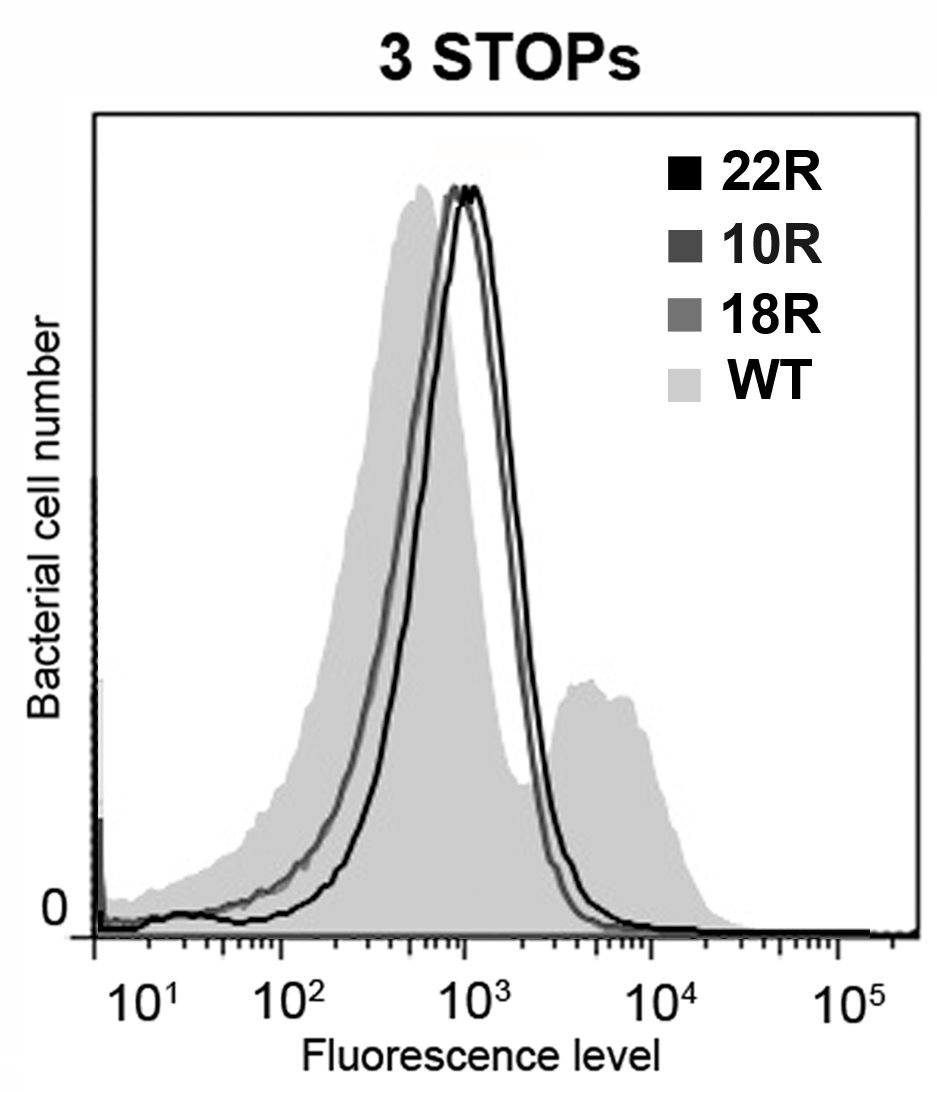

Supplement: Figure S6 — Flow cytometry analysis of UCN34 WT and 3 STOPS mutants with 22R, 18R and 10R GCAGA repeats. All mutants displayed a homogeneous profile when labeled with anti-PilB pAb as compared to the WT profile depicted in grey. (TIF) [file ppat.1003860.s006.tif]

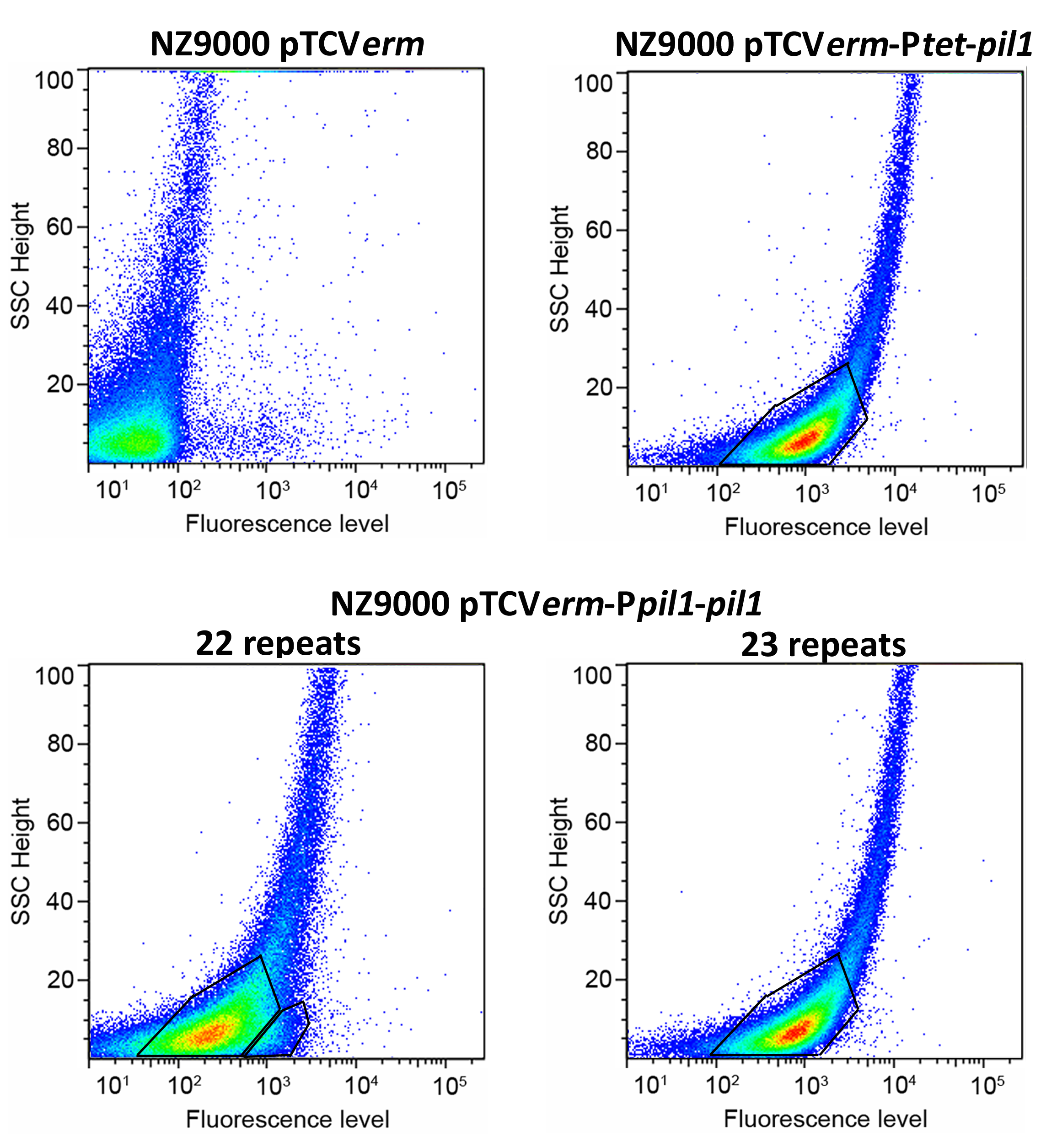

Supplement: Figure S7 — Heterogeneous expression of Pil1 in Lactococcus lactis NZ9000 depends on its 5′ upstream region. Flow cytometry profiles of L. lactis NZ9000 strains expressing pTCVerm, pTCVerm-Ptet-pil1, or pTCVerm-Ppil1-pil1 containing 22 or 23 GCAGA. Note that L. lactis NZ9000 pTCVerm-Ppil1-pil1 with 22 repeats displays a heterogeneous expression of Pil1 with two subpopulations whereas a single population of highly piliated strains was observed with pTCVerm-Ppil1-pil1 containing 23 repeats. As expected, L. lactis cells where pil1 is transcribed from the constitutive promoter Ptet (pTCVerm-Ptet-pil1,) display a single population of highly piliated cells whereas L. lactis expressing the empty vector pTCVerm do not express Pil1. (TIF) [file ppat.1003860.s007.tif]
