# Supplementary material for: Single Cell Stochastic Regulation of Pilus Phase Variation by an Attenuation-like Mechanism
Source: PLoS Pathog. 2014 Jan 16;10(1):e1003860. doi: 10.1371/journal.ppat.1003860 (PMC3894217; doi:10.1371/journal.ppat.1003860)
Supplement: Table S1 — Bacterial strains, plasmids and primers. (DOC) [file ppat.1003860.s008.doc]

**Table S1**. Bacterial strains, plasmids and primers

**Bacterial strains and plasmids**

| **Strains** | **Description** | **Antibiotic marker** | **Reference** |
| --- | --- | --- | --- |
| *Escherichia coli* | | | |
| *DH5α* | F^-^ φ80*lacZ*∆M15 *∆(lac*ZYA-*arg*F)U169 *deo*R *recA*1 *end*A1 *hsd*R17(r_k_^-^ m_k_^+^) *pho*A *sup*E44 *thi*-1 *gyr*A96 *rel*A1 *λ*^-^ | Em | Invitrogen |
| *Streptococcus gallolyticus* | | | |
| UCN34 | MLST 1, infectious endocarditis and colon cancer | Tc | R. Leclercq, (Rusniok et al, 2010) |
| NEM2470 | MLST 1, infectious endocarditis | Tc | R. Leclercq, Caen Hospital |
| NEM2471 | MLST 16, infectious endocarditis | Tc | R. Leclercq, Caen Hospital |
| NEM2472 | MLST 9, infectious endocarditis | Tc | R. Leclercq, Caen Hospital |
| NEM2475 | MLST 14, infectious endocarditis | Tc | R. Leclercq, Caen Hospital |
| NEM2477 | MLST 6, infectious endocarditis | Tc | R. Leclercq, Caen Hospital |
| NEM2479 | MLST 1, infectious endocarditis | Tc | R. Leclercq, Caen Hospital |
| UCN34Δ*pil1* | In frame deletion of the *pil1* genes (*gallo2179*, *gallo2178*, *gallo2177*) | Tc | This work |
| UCN34Δ*term* | In frame deletion of the second strand of the transcriptional terminator | Tc | This work |
| UCN34::A | Mutation of ATG_2_ and of the upstream RBS | Tc | This work |
| UCN34::3STOPs | Addition of 3 stops codon after the repeats | Tc | This work |
| *Streptococcus agalactiae* | | | |
| NEM316 | | Serotype III isolated from neonate blood culture | [Gaillot et al. (1997](http://onlinelibrary.wiley.com/doi/10.1111/j.1365-2958.2006.05190.x/full#b52)) | | --- | --- | |  |  |
| *Lactococcus lactis* | | | |
| NZ9000/pOri23 | *L. lactis* subsp. c*remoris* MG1363, *nisRK*, pOri23 | Em | (Danne *et al*., 2011) |
| NZ9000/pOri23Ω*pil1* | *L. lactis* subsp. *cremoris* MG1363, *nisRK*, pOri23Ω*pil1* | Em | (Danne *et al*., 2011) |
| **Plasmids** | | | |
| pTCV*erm* | Em, Km, Mob^+^ (IncP); *oriR* pACYC184; *oriR* pAMβ1  (low copy number); MCS *lacZ*α^+^ | Em | (Poyart *et al.*, 2001) |
| pTCV*lac* | Em, Km, Mob^+^ (IncP); *oriR* pACYC184; *oriR* pAMβ1  (low copy number); MCS for transcriptional fusion  with a promoterless *spoVG-lacZ* gene | Em | (Poyart and Trieu-Cuot, 1997) |
| pOri23 | Em*, oriR*s pUC18 et pIP501 (Gram- / Gram+ shuttle vector) | Em | (Que *et al*., 2000) |

**Primers**

| **Name** | **Sequences 5’→ 3’^a^** | **Amplified fragment** |
| --- | --- | --- |
| **Primer extension** | | |
| CD35 | AGTGCTTTAGATAAGCGATGTTTGG | 654 pb |
| CD37 | GCTTCCTAAAATTTATTATCCCTTCTCC | 654 pb |
| **Beta-galactosidase assay** | | |
| **pTCVlac-+1_(-390pb)_** | | |
| CD155 | AAGGATCCTTTAATATACAATTGTACTCA | 406 pb |
| Prom79Eco | GGGGGAATTCCTCTACTTGAATAATGTAATATTTCTG | 406 pb |
| **pTCVlac-+1_(-130pb)_** | | |
| CD138 | CAGAATTCTTGTTCGTTCTTTTTGAGTAC | 128 pb |
| CD139 | TCGGATCCTTTTATACTAATATCAAAGCC | 128 pb |
| **Mutants construction** | | |
| **UCN34Δ*pil1*** | | |
| CDΔ*pil1*-5' | CGGTGTCCTCAACACACCAAGGGAG | 942 pb |
| CDΔ*pil1*-5'c | GTAATCTGCGGTAAACATTTGGATCCCCGGGTACCACTTTATCACTCCCTTT | 942 pb |
| CDΔ*pil1*-3'c | AGGGAGTGATAAAGTGGTACCCGGGGATCCAAATGTTTACCGCAGAT | 977 pb |
| CDΔ*pil1*-3' | CCCAGACCTACCATATGAGCCGTGAC | 977 pb |
| **UCN34Δ*term*** | | |
| CD156 | TGGAATTCAAAGCGAGAAATGTCCAC | 799 pb |
| CD167 | CCCTTTTATACTAATATCAAAGCCATCTAAATACACTAAAAAAAC | 799 pb |
| CD166 | GTTTTTTTAGTGTATTTAGATGGCTTTGATATTAGTATAAAAGGG | 868 pb |
| CD106 | GGACGGATCCTCTTAAACTCTTCTAAGGC | 868 pb |
| **UCN34::A** | | |
| CD103 | TCGAATTCAATAGTGAAAAATCTGGTAA | 908 pb |
| CD168 | GCTGGTGGTAGCAAGAGGTTACATTCTTGTCAAC | 908 pb |
| CD169 | AGAATGTAACCTCTTGCTACCACCAGCAGAGCAG | 856 pb |
| CD159 | GTGGATCCTTGATGTTCCTGTTCCTG | 856 pb |
| **UCN34::3STOPs** | | |
| CD156 | TGGAATTCAAAGCGAGAAATGTCCAC | 740 pb |
| CD180 | CAATTGTACTCAAAAAGAATCAGTTAGCTATCTGCTCTGCTCT | 740 pb |
| CD181 | GAGCAGAGCAGATAGCTAACTGATTCTTTTTGAGTACAATTG | 787 pb |
| CD175 | TAGGATCCACCTAACCCTATTAGTATCGC | 787 pb |
| **Complementation** | | |
| **pTCV*erm*-P*pil1-pil1*** | | |
| Prom79Eco | GGGGGAATTCCTCTACTTGAATAATGTAATATTTCTG | 541 pb |
| Prom79Bam | ACGGGGATCCTTTATTAACATAATTATCCATTAGCAC | 541 pb |
| CD*pil1*-Bam | ATTAGGGATCCAGGGAGTGATAAAGTGGTTGCT | 4752 pb |
| CD*pil1*-Xba | GCATTCTAGATACCGTCGCCCAAACAGT | 4752 pb |
| **Transcriptional analyses** | | |
| 1 | CTCAAAAAGAACGAACAAACTATCTGC | 157 pb |
| 2 | agaatgtaaggagttgctacca | 157 pb |
| 3 | gcttcctaaaatttattatcccttctcc | 117 pb |
| 4 | aaagggagtgataaagtggtt | 117 pb |
| gallo2178-fwd | ACTGTTGAGAACGGTGGTAGTGGA | 125 pb |
| gallo2178-rev | GTTTGACCAGCTGTAGTGATGCCA | 125 pb |
| gallo2179-fwd | CACTATTGAGGTCACCTGGTCGAT | 173 pb |
| gallo2179-rev | CCCACCCTGATACATTTTCCATTG | 173 pb |
| gallo16SRNA-fwd | CAGGTCTTGACATCCCGATGCTAT | 169 pb |
| gallo16SRNA-rev | CGCTAGAGTGCCCAACTGAATGAT | 169 pb |
| tanA-Fwd | AAAAGCGGCTGTTAGGTGGCTTAG | 132 pb |
| tanA-Rev | TCTGCTCGATTTCCTGTGCTACCT | 132 pb |

a : Restriction sites are in bold.
